# Thrombosis-related circulating miR-16-5p is associated with disease severity in patients hospitalised for COVID-19

**DOI:** 10.1080/15476286.2022.2100629

**Published:** 2022-08-07

**Authors:** Ceren Eyileten, Zofia Wicik, Sérgio N. Simões, David C. Martins-Jr, Krzysztof Klos, Wojciech Wlodarczyk, Alice Assinger, Dariusz Soldacki, Andrzej Chcialowski, Jolanta M. Siller-Matula, Marek Postula

**Affiliations:** a Department of Experimental and Clinical Pharmacology, Medical University of Warsaw, Center for Preclinical Research and Technology CEPT, Warsaw, Poland; b Genomics Core Facility, Centre of New Technologies, University of Warsaw, Warsaw, Poland; c Center for Mathematics, Computing and Cognition, Federal University of ABC, Santo André Brazil; d Department of Informatics, Federal Institute of Espírito Santo, Serra, Brazil; e Department of Infectious Diseases and Allergology - Military Institute of Medicine, Warsaw, Poland; f Department of Vascular Biology and Thrombosis Research, Center of Physiology and Pharmacology, Medical University of Vienna, Austria; g Department of Clinical Immunology, Medical University of Warsaw, Warsaw, Poland; h Department of Internal Medicine II, Division of Cardiology, Medical University of Vienna, Vienna, Austria

**Keywords:** ACE2, microRNAs, miRNA, bioinformatics analysis, in silico prediction, SARS-COV-2

## Abstract

SARS-CoV-2 tropism for the ACE2 receptor, along with the multifaceted inflammatory reaction, is likely to drive the generalized hypercoagulable and thrombotic state seen in patients with COVID-19. Using the original bioinformatic workflow and network medicine approaches we reanalysed four coronavirus-related expression datasets and performed co-expression analysis focused on thrombosis and ACE2 related genes. We identified microRNAs (miRNAs) which play role in ACE2-related thrombosis in coronavirus infection and further, we validated the expressions of precisely selected miRNAs-related to thrombosis (miR-16-5p, miR-27a-3p, let-7b-5p and miR-155-5p) in 79 hospitalized COVID-19 patients and 32 healthy volunteers by qRT-PCR. Consequently, we aimed to unravel whether bioinformatic prioritization could guide selection of miRNAs with a potential of diagnostic and prognostic biomarkers associated with disease severity in patients hospitalized for COVID-19. In bioinformatic analysis, we identified EGFR, HSP90AA1, APP, TP53, PTEN, UBC, FN1, ELAVL1 and CALM1 as regulatory genes which could play a pivotal role in COVID-19 related thrombosis. We also found miR-16-5p, miR-27a-3p, let-7b-5p and miR-155-5p as regulators in the coagulation and thrombosis process. *In silico* predictions were further confirmed in patients hospitalized for COVID-19. The expression levels of miR-16-5p and let-7b in COVID-19 patients were lower at baseline, 7-days and 21-day after admission compared to the healthy controls (p < 0.0001 for all time points for both miRNAs). The expression levels of miR-27a-3p and miR-155-5p in COVID-19 patients were higher at day 21 compared to the healthy controls (p = 0.007 and p < 0.001, respectively). A low baseline miR-16-5p expression presents predictive utility in assessment of the hospital length of stay or death in follow-up as a composite endpoint (AUC:0.810, 95% CI, 0.71–0.91, p < 0.0001) and low baseline expression of miR-16-5p and diabetes mellitus are independent predictors of increased length of stay or death according to a multivariate analysis (OR: 9.417; 95% CI, 2.647–33.506; p = 0.0005 and OR: 6.257; 95% CI, 1.049–37.316; p = 0.044, respectively). This study enabled us to better characterize changes in gene expression and signalling pathways related to hypercoagulable and thrombotic conditions in COVID-19. In this study we identified and validated miRNAs which could serve as novel, thrombosis-related predictive biomarkers of the COVID-19 complications, and can be used for early stratification of patients and prediction of severity of infection development in an individual.**Abbreviations:** ACE2, angiotensin-converting enzyme 2AF, atrial fibrillationAPP, Amyloid Beta Precursor ProteinaPTT, activated partial thromboplastin timeAUC, Area under the curveAβ, amyloid betaBMI, body mass indexCAD, coronary artery diseaseCALM1, Calmodulin 1 geneCaM, calmodulinCCND1, Cyclin D1CI, confidence intervalCOPD, chronic obstructive pulmonary diseaseCOVID-19, Coronavirus disease 2019CRP, C-reactive proteinCV, CardiovascularCVDs, cardiovascular diseasesDE, differentially expressedDM, diabetes mellitusEGFR, Epithelial growth factor receptorELAVL1, ELAV Like RNA Binding Protein 1FLNA, Filamin AFN1, Fibronectin 1GEO, Gene Expression OmnibushiPSC-CMs, Human induced pluripotent stem cell-derived cardiomyocytesHSP90AA1, Heat Shock Protein 90 Alpha Family Class A Member 1Hsp90α, heat shock protein 90αICU, intensive care unitIL, interleukinIQR, interquartile rangelncRNAs, long non-coding RNAsMI, myocardial infarctionMiRNA, MiR, microRNAmRNA, messenger RNAncRNA, non-coding RNANERI, network-medicine based integrative approachNF-kB, nuclear factor kappa-light-chain-enhancer of activated B cellsNPV, negative predictive valueNXF, nuclear export factorPBMCs, Peripheral blood mononuclear cellsPCT, procalcitoninPPI, Protein-protein interactionsPPV, positive predictive valuePTEN, phosphatase and tensin homologqPCR, quantitative polymerase chain reactionROC, receiver operating characteristicSARS-CoV-2, severe acute respiratory syndrome coronavirus 2SD, standard deviationTLR4, Toll-like receptor 4TM, thrombomodulinTP53, Tumour protein P53UBC, Ubiquitin CWBC, white blood cells

## Graphical Abstract

**Figure:**
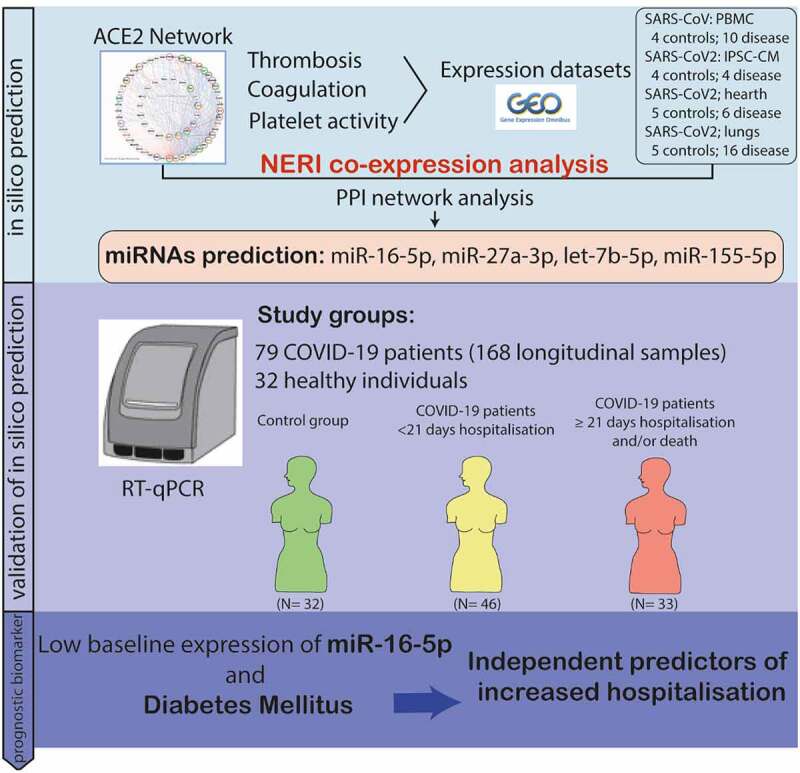

## 1. Introduction

Coronavirus disease 2019 (COVID-19) is caused by severe acute respiratory syndrome coronavirus 2 (SARS-CoV-2) and is associated with increased risk of mortality and adverse cardiovascular (CV) events especially among patients with underlying cardiovascular diseases (CVD) [1,2].

Key mechanisms which may drive the pathophysiology of multi-organ injury secondary to infection with SARS-CoV-2 include direct viral toxicity, thrombosis and inflammation, leading to thromboinflammation which is simultaneous and co-localized activation of thrombotic and inflammatory response [3,4]. Thromboinflammation is associated with dysregulation of normal anti-thrombotic and anti-inflammatory functions of endothelial cells and negatively influences haemostasis, which is especially dangerous in microvasculature, where microthrombi deposition can occur [5,6]. In line, patients with severe COVID-19 present with microvascular thrombosis and haemorrhage. This was associated with lung pathology presenting as extensive alveolar and interstitial inflammation which could be suggestive for the assessment of the case fatality [7]. Autopsy studies have revealed the presence of thromboinflammation within the pulmonary capillary vasculature. Moreover, it should be noted that patients with COVID-19 are more prone to develop pulmonary embolism, deep vein thrombosis, arterial thrombosis and intracatheter thrombosis, which is also negative for survival prognosis [8,9].

Early in the pandemic, hospitalized patients with COVID-19 were often observed to have changes in the levels of coagulant biomarkers, including fibrinogen, D-dimer and activated partial thromboplastin time (aPTT), and routine measurement of these biomarkers was recommended [10–12]. However, a number of other markers of coagulation have emerged that have helped to refine our understanding of the thrombotic signature of COVID-19 [13]. Currently there is a need for identification of biomarkers which would allow early identification of patients in risk of thrombosis. MicroRNAs (miRNAs, miRs) are a class of small, endogenous, non-coding RNA (ncRNA) molecules that have such a potential. They play an important role in many biological processes and regulate the expression of approximately 60% of the mammalian protein coding genes [14]. MiRNAs act primarily by binding to complementary regions of messenger RNA (mRNA), leading to repression of its translation or even induction of its degradation [15]. Various miRNAs, as well as their target genes are implicated in the complex pathophysiology of coagulation and CVDs [16]. Thus, they may be useful for diagnosis, prognosis and as a potential therapeutic strategy in multiple pathologies [1,15,17–25]. There is also growing evidence from epidemiological studies and animal models suggesting that the expression level of miRNAs not only regulates coagulation and haemostatic factors but is dysregulated in venous thromboembolism (VTE) [26]. One of the case-control studies identified 9 differentially expressed (DE) miRNAs (hsa-miR-4451, hsa-miR-942-3p, hsa-miR-8063, hsa-miR-3132, hsa-miR-3118, hsa-miR-105-5p, hsa-miR-891a-5p, hsa-miR-200a-5p, and hsa-miR-6832-3p) in the blood of colorectal cancer patients who developed VTE compared to controls in this pilot study [27]. Other diagnostic biomarkers for PE include miR-134 [28], miR-1233 [29] and miR-28-3p [30]; while diagnostic biomarkers for DVT include miR-582, miR-195, miR-532 [31], miR-424-5p, miR-136–5p [32], and miR-320a, miR-320b [33]. Taken together, due to their stability and regulation of a high number of disease-related genes miRNAs may be relevant not only as biomarkers for different kinds of thrombosis but could also be involved in the pathogenesis of thrombosis and, therefore, be potential therapeutic targets.

Implementation of bioinformatics tools can reveal interactions between genes and their non-coding regulators, i.e. miRNAs, which may help to understand the pathomechanisms of disease development [1,34]. A biggest advantage of network medicine and systems biology approach is the ability to identify novel, key regulators of the pathological processes including investigation of the COVID-19 related changes in coagulation processes. Barabási et al. summarized a series of hypotheses and principles (Network Medicine Hypotheses) which link topological properties of Protein-Protein Interaction (PPI) networks to biological processes [35]. Those hypotheses are often used to prioritize candidate genes associated with the analysed disease. We highlight three hypotheses (i) disease module hypothesis: gene products associated with the same disease phenotype tend to form a cluster in the PPI network; (ii) network parsimony: disease pathways often coincide with shortest paths between known disease genes; (iii) local hypothesis: gene products associated with similar diseases are likely to strongly interact with each other [35]. In this way, we used an innovative NERI algorithm [36] which integrates the interactome data with co-expression networks, enabling us to focus on the signalling cascade between ACE2 and genes associated with thrombosis and coagulation-related processes. By assuming some Network Medicine hypothesis (such as: network parsimony and disease modularity), the method explored the neighbourhood of a gene set (seeds) by choosing the smallest paths possessing more coexpressed genes with the seeds. As output, the method returns two subnetworks (modules): control and disease subnetworks, for which the method ranks genes and interactions (edges) according to their topological importances in both subnetworks (score X) or to their topological alterations between the subnetworks (score Δ’, also called S internally by the algorithm implementation). This approach enabled us to discover a cluster of party hub genes [37,38], also called ‘bottleneck regulators’, with corroborating signals across transcript expression and protein-protein interaction data, causing to pathological alterations in coagulation processes even when such regulators show little or no changes in expression between control and disease conditions.

MiRNAs have been studied as diagnostic and prognostic biomarkers for many diseases including myocardial infarction, liver failure, sepsis, or ischaemic stroke [39,40]. Whilst protein-based biomarkers for COVID-19 have been highly studied [41,42], however, limited number of studies aimed to analyse preselected circulating miRNAs in COVID-19 patients thus far [43–45]. Lately, the strong association of cardiometabolic miRNAs with COVID-19 severity and mortality has been shown and combinations of miRNAs improved classification performance of established markers for severity and mortality of COVID-19 [45].

In line with previous publications, in the current study, we have precisely selected analysed miRNAs based on bioinformatic analysis including the top thrombosis and coagulation-related miRNA (miR-16, let-7b, miR-27a, miR-155) and aimed to unravel whether bioinformatic prioritization could guide selection of miRNAs with a potential as diagnostic and prognostic biomarkers associated with disease severity in patients hospitalized for COVID-19.

## 2. Methods

### 2.1. Bioinformatics analysis: miRNA targets prediction, data filtering, and visualization as interaction networks

#### 2.1.1. Gene lists selection

We downloaded the following lists of genes: 98 genes associated with thrombosis term from Disgenet database (https://www.disgenet.org/browser/0/1/0/C0040053/); 62 genes associated with thrombosis term from Malacards database; 224 genes related to coagulation and 341 genes related to platelet activity extracted by BiomartR R package from Gene Ontology database. The 68 genes involved in the ACE2 network were obtained from a previous publication [1]. The gene symbols were unified using the NCBI annotation file.

#### 2.1.2. Tissue specific expression

In order to identify genes with expression in the CV system, we mined Tissues 2.0 database [46]. For further PPI interactions analyses leading to seed gene selection we selected genes with expression confidence cut-off at least 2 in Blood, Heart, Pericyte or CV system. For pre-selection of miRNAs we also used the ones expressed with high confidence in blood and above mentioned tissues.

#### 2.1.3. Interactome analysis and visualization

All visualizations of the networks were done using Cytoscape v3.8.2 [47]. Protein-protein interactions (PPI) between analysed genes were obtained from StringApp v 1.7.0, using default confidence cut-off (≥0.4) [48].

#### 2.1.4. Expression datasets analysis

We downloaded three expression datasets from the Gene Expression Omnibus (GEO) database: 1) Peripheral blood mononuclear cells (PBMCs) isolated from SARS patients and controls (GSE1739) [49]; 2) Human induced pluripotent stem cell-derived cardiomyocytes (hiPSC-CMs) infected with SARS-CoV2 vs control cultures (GSE150392)[50]; 3) Autopsies of SARS-CoV-2 infected tissues from which we selected datasets related to Lungs, Hearts and control samples (GSE150316)[51]. We performed differential expression analysis for each dataset using a linear regression model (GSE1739, GSE150316) or Mann Whitney test (GSE150392). As a differentially expressed (DE) gene we selected those which had FDR adjusted p-value <0.05.

#### 2.1.5. PPI network analysis with NERI method

To identify top genes which could play a role in COVID-19-related thrombosis but are not necessarily DE we performed co-expression analysis using network-medicine based integrative approach (NERI) algorithm [36] and three sets of seed genes. The selection of the seeds is described in the results section (Selection of the ACE-2 related coagulation seed genes). The NERI method returns a subnetwork representing the neighbourhood of a gene set by selecting the shortest paths between all pairs of seeds with the most coexpressed genes along the paths. This method is independently applied for two conditions (control and disease), thus obtaining the control and disease subnetworks (modules). The method outputs two scores X and Δ’ (S) regarding the topological analysis of the two resulting subnetworks (control and disease). The first one (X) prioritizes genes with party hub features [37,38] in both modules, possessing high topological centrality and, at the same time, high co-expression relative to the seed genes. The second one (Δ’) prioritizes the most topologically altered genes and edges between the two conditions [36]. Each analysis provided 100 best nodes and 500 best edges. In further steps for constructing the visualization of the interaction networks: i) we selected genes connected by best edges and best edges associated with best nodes; ii) genes which appeared in at least 5 out of 8 analysed datasets using coagulation related seeds.

#### 2.1.6. Selection of the top genes based on co-expression network analysis using NERI

In order to identify the top genes which could serve as signalling ‘bottleneck’ in COVID-19 we performed interaction analysis using four different expression datasets: PBMC from COVID-19 patients, hiPSC-CMs infected with SARS-CoV2, heart and lung tissues from deceased COVID-19 patients. Each dataset was analysed using NERI algorithm [36], which integrated protein-protein interactome data with expression data using so-called seed-genes, enabling us to focus on the part of the network related to: (*i) ACE2 interactome, (ii) interaction of ACE2-related genes with coagulation-related genes, (iii) coagulation related genes with high proximity to ACE2 interactome*. The algorithm ranked genes based on their role as hub genes connecting multiple signalling pathways and disease-related changes of expression in neighbouring genes with correlated expression changes. In a further step, we overlapped all 12 outputs (4 expression datasets, 3 seeds each) to identify clusters of the top genes and interactions regulated by coronaviruses. For further analyses we used only results for the following sets of seeds: (i) interaction of ACE2-related genes connected coagulation-related genes; (ii) coagulation related genes with high proximity to ACE2 interactome. Simultaneously, we performed enrichment analysis to verify whether coagulation-related processes were also affected by this virus and ACE2 signalling. We also performed visualization of the interaction network between top genes. In the final step, we identified the top microRNAs (miRNA) which targeted the highest number of the top genes as well as the highest number of DE genes (affected by the disease) across analysed datasets.

#### 2.1.7 miRNA target prediction:

On all steps of bioinformatic analyses we used the R package ‘wizbionet’ https://github.com/wizbionet/wizbionet/blob/master/doc/vignette_wizbionet.md [52]. To identify miRNAs regulating DE genes we used a topmiRNA_toptarget function based on the multiMiR R package [53]. We looked for the −3p and −5p mature version of each miRNA identified by multiMiR and their targets among DE genes and coagulation/thrombosis related genes. Then we searched the top 10% hits among all conserved and non-conserved target sites in 14 target prediction databases.

#### 2.1.8. Enrichment analysis

Enrichment analysis is a computational method for increasing the likelihood to identify the most significant biological processes related to the study. Enrichment analysis of the networks was done using StringApp and EnrichR database[54], using the Hypergeometric test with Benjamini and Hochberg correction, while the reference was the human genome. For all enrichment analyses, the significance cut-off was set to BH adjusted p-value ≤0.05.

### 2.2 Ethics statement

The study was conducted with the Declaration of Helsinki [55]. The study protocol was approved by an ethical committee. Written informed consent form was obtained from all participants, or their legal representatives.

### 2.3. Study group

This was an observational study which included 79 COVID-19 patients admitted to the Clinic of Internal Medicine, Pneumonology, Allergology and Clinical Immunology at the Military Institute of Medicine in Warsaw. Patients aged 18 years or older with a positive nasopharyngeal swab PCR test for SARS-CoV-2 were recruited during the third pandemic wave in Poland (January-May 2021), who were admitted to the hospital based on clinical presentation including severe dyspnoea requiring oxygen therapy and monitoring on intensive care unit (ICU) for at least 24 hours. Blood samples of COVID-19 patients were collected at three different time points, including the day of admission, 7-days and 21-days after admission. The patients were divided according to the hospitalization length of stay (>21 days) and/or death in follow-up as a composite endpoint. Thirty two age and sex matched SARS-CoV-2 infection-free participants were sampled in out-patients clinic.

Comprehensive demographic, clinical, pharmacological and laboratory data were abstracted manually from the electronic medical records. All blood samples were collected as whole blood by using Tempus™ Blood RNA Tube (Applied Biosystems). All specimens were immediately aliquoted, frozen and stored at −80°C. Frozen sample was thawed once only to prevent repeated freeze-thaw cycles.

### 2.4. RNA preparation, detection, and quantification of miRNAs by quantitative PCR

Total RNA was extracted using Tempus™ Spin RNA Isolation Kit (Invitrogen) from 9 ml whole blood. Subsequently, the obtained RNA template was subjected to a reverse transcription reaction using the TaqMan miRNA Reverse Transcription kit (ABI, California, USA) according to guidelines provided by the manufacturer. Afterwards, miRNA expressions were detected by quantitative polymerase chain reaction (qPCR) using TaqMan miRNA Advanced Assay kits (ABI, California, USA, catalogue number A25576, assay ID; 477860_mir; 478576_mir; 478384_mir; 477927_mir) for the corresponding miRNAs on a CFX384 Touch Real-Time PCR Detection System (BioRad Inc. Hercules, California, USA). During the RNA extraction phase cel-miR-39 was spiked-in as an exogenous control and all qRT-PCRs were normalized to their corresponding cel-miR-39. Mean values of all reactions – performed in triplicate – were used in statistical analysis as previously described [56,57]. MiRNA expressions were expressed as 2-ΔCT [miRNA expression – cel-miR-39 expression] [56,58–60], then log-10 transformed for statistical analysis.

### 2.5 Statistical analysis

All results for categorical variables were presented as a number and percentage. Continuous variables were expressed as mean ± standard deviation (SD) or median and interquartile range (IQR), depending on the normality of distribution assessed by means of a Shapiro–Wilk test. The Student t-test or the Mann-Whitney test for unpaired samples, and Wilcoxon test for paired samples, were applied depending on the normality of the distribution. One-way Anova or Kruskal-Wallis tests were used for more than two groups comparison based on the distribution of the data. Receiver operating characteristic (ROC) analysis was performed to assess the predictive value of miR-16-5p for increased hospital length of stay or death in follow-up as a composite endpoint. To determine independent variables affecting increased hospital length of stay or death we applied multivariate logistic regression analysis. For this purpose, we included the following variables: low miR-16-5p (>6.1 expression based on Log-transformed data), age (years), male sex, BMI >30, hypertension, diabetes mellitus, smoking and coronary vascular disease. All tests were two-sided with the significance level of p < 0.05. Calculations were performed using SPSS version 22.0 (IBM Corporation, Chicago, USA). Based on a 76% increased hospitalization in the low baseline miR-16-5p group as compared to 24% increased hospitalization in the high baseline miR-16-5p group, we calculated that with 79 patients, our analysis had 99% power with a two-sided alpha value of < 0.05.

## 3. Results

### 3.1. Bioinformatic analysis results

**Figure 1.**
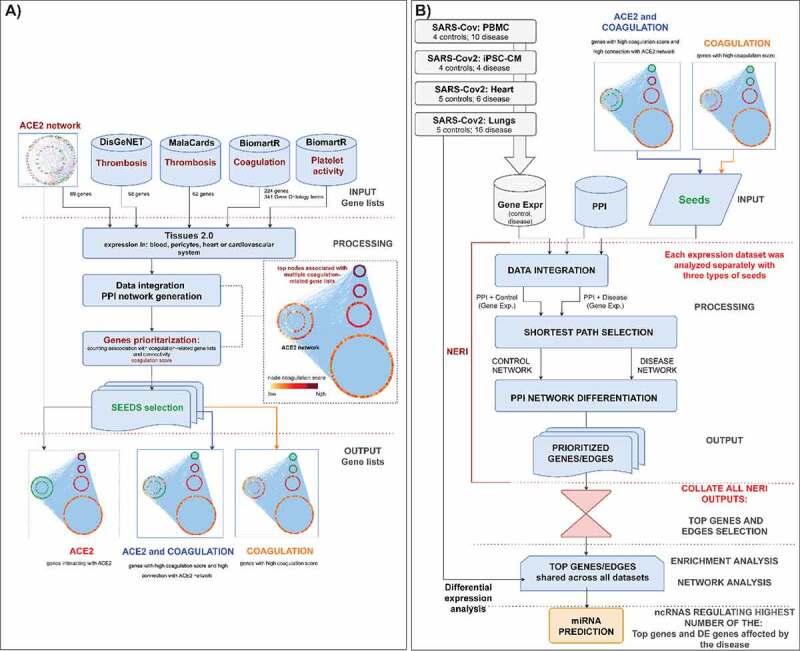
**A) Bioinformatic workflow of the ACE2 and thrombosis-related seed genes selection for the integration of PPI interactome data with co-expression networks by NERI algorithm**. This analysis enabled us to focus on a specific part of the network in this case: *gradual signalling cascade between ACE2 and genes associated with coagulation-related processes*. Seed genes are marked in green. Nodes outside of the ACE2 network were sorted using group circular layout based on the number of occurrences on four thrombosis and coagulation- related gene lists. Node colours were related to so-called ‘coagulation score’ calculated based on the occurrences on four gene lists and expression in four cardiovascular-system related tissues. **B) Bioinformatic workflow leading to the identification of the miRNAs regulating the coagulation process in coronavirus infection.**

In this study we aimed to, based on co-expression analysis using NERI algorithm and set seed genes, to identify clusters of hub genes [37,38]. Those genes, also called ‘bottleneck regulators’ through corroborating signals across transcript expression and protein-protein interaction data, would lead to pathological changes in coagulation processes even when such regulators show little or no changes in expression between control and disease conditions. Then based on those results we identified top non-coding regulators of the interaction between ACE2 interaction networks which could be affected by SARS-CoV-2 infection and coagulation process. Identified in this study miRNAs were shared regulators of ACE2 network and coagulation-related genes between PBMCs, heart, lungs and cardiomyocytes infected with coronaviruses. The workflow of the analysis is shown on the (Fig. 1A,B).

#### 3.1.1. Identification of the ACE2 related coagulation seed genes

In order to select the best seed genes for further co-expression analysis using NERI we used 69 genes identified in our previous study as involved in ACE2 interaction network [1] and further extended it for genes related to thrombosis and coagulation. For each gene we calculated the so-called coagulation score based on its expression in the CV system, presence in expression datasets, and presence in disease-related gene lists (thrombosis term in Malacards/DisgeNet); and finally its connection with the ACE2 network. For further analyses we selected three genesets: i) **ACE2**- 68 genes interacting with ACE2; ii) **ACE2 and coagulation**- 53 genes with high coagulation score and high connectivity with ACE2 network; iii) **Coagulation**- 40 genes with high coagulation score (≥7) with high proximity to ACE2 interactome. All genes used in seed selection analysis are shown in the **Supplementary Figure 1** and are available as a Cytoscape network file (Supplementary file 1).

#### 3.1.2. Top coagulation-related genes affected by coronavirus infections

**Figure 2.**
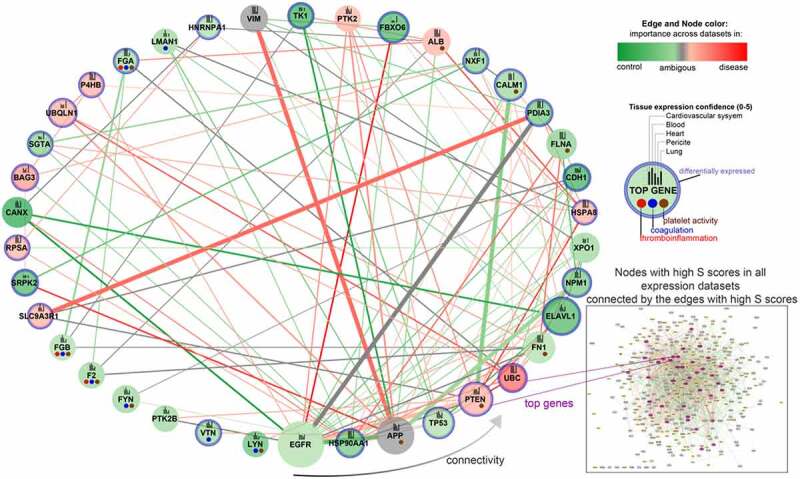
**Top nodes identified by NERI algorithm as associated with coagulation networks in SARS and SARS-CoV-2 infections**. The edge size is related to the number of occurrences in the top 30% of strongest regulated edges across analysed datasets (SARS, cardiomyocytes, lungs, heart) and two types of seeds ***‘ACE2 and coagulation’*** and ‘**coagulation**’ (8 gene lists in total). The node and edge sizes are associated with the importance in signal flow within the network for the analysed expression datasets (12 in total), which does not necessarily relate to upregulation in expression. If node/edge was enhanced according to NERI in higher number of disease-related datasets, it has red colour and has high importance in disease, if in control-related dataset, it is green and has decreased importance in disease. Grey colour means disease or control rate was the same in all datasets. The node size is associated with the sum of Δ’ (S) scores obtained from NERI. The higher the S score, the more important the role of a given gene in the analysed network. To show the tissue-specific expression level of each gene we added bars on each node reflecting their expression confidence (0–5) using the data obtained from the Tissues 2.0 database. ***EGFR, ELAVL1 and APP*** were identified across all 12 analysed gene lists (including three sets of seeds) as ***the top regulators of the thrombosis-related networks***. DE genes in expression datasets have blue borders.

Coexpression analysis using NERI across all analysed datasets and seeds, allowed us to found several top genes which could play a pivotal role in COVID-19 related thrombosis, such as **EGFR, HSP90AA1, APP, TP53, PTEN, UBC, FN1, ELAVL1 and CALM1 (**Fig. 2) (Abbreviations: EGFR, Epithelial growth factor receptor; HSP90AA1, Heat Shock Protein 90 Alpha Family Class A Member 1; APP, Amyloid Beta Precursor Protein; TP53, Tumour protein P53; PTEN, phosphatase and tensin homolog; UBC, Ubiquitin C; FN1, Fibronectin 1; ELAVL1, ELAV Like RNA Binding Protein 1; CALM1, Calmodulin 1 gene).

**Figure 3.**
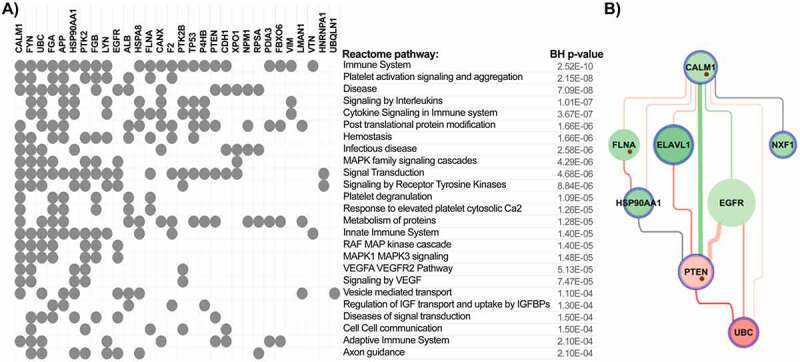
**A) Top 25 enriched pathways associated with top nodes identified by NERI algorithm as important in coagulation networks in SARS and SARS-CoV-2 infections**. Grey dots indicate whether a gene is present within significantly enriched signalling pathways. It is worth noticing that CALM1 was present in the highest number of signalling pathways as an important ACE2 interactor. The top interactors with highest number of associated pathways were ordered in decreasing manner from left to right. **B) Alterations between CALM1 interactors in COVID-19**. Green colour is associated with loss of importance in disease which can lead to switching the signalling onto neighbouring nodes. Red colour is associated with increased importance of the node/edge in disease, leading to increase of signalling in a given part of the network.

The detailed workflow of top genes selection is described in the Methods section. Among the top genes identified by enrichment analysis as playing a key role in signalling pathways associated with coagulation-related interaction networks in COVID-19, we identified **CALM1** and enhancement of its connection with **PTEN and UBC (**Fig. 3A,B).

#### 3.1.3 Pathway enrichment analysis identified CALM1 as the most potent regulatory gene

In order to identify thrombosis-related pathways affected by coronavirus infection we performed enrichment analysis of the top genes. This analysis revealed that the highest number of the top genes was associated with pathways related to immune system and platelet activation signalling and aggregation. We also observed a strong enrichment of disease, signalling by interleukins and cytokines (Fig. 3A). CALM1 was identified in this analysis as a gene connected to the highest number of significantly overrepresented pathways. Closer analysis of its interaction network showed that PTEN and its interaction with CALM1 was stronger in control samples than in coronavirus samples, while interactions with ELAVL1, EGFR and UBC were stronger in the disease (Fig. 3B).

#### 3.1.4. Identification of the miRNAs regulating ACE2 and coagulation related interaction networks and DE genes affected in COVID-19

In order to identify miRNAs which play a role in ACE2-related thrombosis in SARS-CoV2 infection we screened four lists of DE genes (SARS, cardiomyocytes, heart and lungs), as well as DE coagulation genes from those datasets. Next, we looked for miRNAs regulating the highest number of top NERI nodes and top NERI targets associated with coagulation. Top miRNAs were defined as regulating the top 30% of genes within each category (all DE genes, coagulation DE genes, top NERI nodes, top NERI nodes associated with ACE2 related coagulation). We performed target predictions for the 1416 miRNAs showing any expression confidence in the cardiovascular system according to the Tissues 2.0 database. In this analysis we identified 34 pre-miRNAs which targeted the highest number of ‘bottleneck regulators’ associated with coagulation shared across four COVID-19 related datasets, and the highest number of DE genes in those datasets (Table 1). Additionally in this table we listed which coagulation-related genes with high scores in NERI analysis, including 13 coagulation genes from Fig. 1, are regulated by the top miRNAs. Among the identified miRNAs that regulated ACE2-coagulation related interaction networks, we have found 8 pre-miRNAs, namely miR-16, miR-27a, miR-34a, let-7b, miR-155, miR-23b, miR-374a and miR-128, that shared the highest number of the top genes identified using NERI and DE genes related to coronavirus infection. In our previous *in silico* prediction analysis we found miR-16-5p and miR-27a-3p as miRNAs regulating ACE2 networks [1]. Moreover another *in silico* analysis by Jafarinejad-Farsangi et al. [61] found miR-16-5p and let-7b as targeting SARS-CoV-2 induced differentially expressed genes and miR-155 was predicted related to SARS‑CoV‑2‑induced cytokine storm [62]. Therefore, out of eight miRNAs that were found in the current analysis, we selected particularly four the most promising miRNAs (i.e. miR-27a, miR-16, let-7b and miR-155), and further validated their expression by qRT-PCR in patients hospitalized due to COVID-19.

**Table 1.** **The top miRNAs which regulate ACE2-Coagulation related interaction networks**. Top miRNAs were identified based on their regulation of highest number of the top differentially expressed genes (DE) across all analysed datasets, top DE genes associated with coagulation across all analysed datasets, top genes identified by NERI analysis, and top genes identified by NERI analysis and also involved in coagulation process. Additionally we showed whether miRNA was targeting ELAVL1 or EGFR gene (not present on complete coagulation gene list). Mature miRNAs marked with bold font were examined using qPCR.

Pre-miR	Mature miRNA	Top all DE genes	Top DE genes related to coagulation	Top NERI	Top NERI: ACE2-related coagulation	Top in analyses:	NERI top coagulation targets	ELAVL1	EGFR
hsa-miR-16	**hsa-miR-16-5p**	**+**	**+**	**+**	**+**	**4**	APP| CALM1| CAV1| CBL| COL1A1| F2| FGB| FLNA| FN1| LMAN1| LRP1| PROS1| TFPI| VTN| YWHAZ	-	**+**
hsa-miR-27a	**hsa-miR-27a-3p**|hsa-miR-27a-5p	**+**	**+**	**+**	**+**	**4**	ALB| CAV1| CBL| COL1A1| FN1| LRP1| PLG| PROS1| PTPN11| SERPING1| TFPI| YWHAZ	**+**	**+**
hsa-miR-34a	hsa-miR-34a-3p|hsa-miR-34a-5p	**+**	**+**	**+**	**+**	**4**	APP| CALM1| COL1A1| FN1| FYN| LMAN1| PTEN| PTPN11| TFPI| YWHAZ	**+**	**+**
hsa-let-7b	hsa-let-7b-3p|**hsa-let-7b-5p**	**+**	**+**	**+**	**+**	**4**	APP| CALM1| CBL| COL1A1| F2| FLNA| FN1| LRP1| LYN| PROS1| TFPI| YWHAZ	**+**	**+**
hsa-miR-155	hsa-miR-155-3p|**hsa-miR-155-5p**	**+**	**+**	**+**	**+**	**4**	ALB| APP| CALM1| CAV1| CBL| COL1A1| F2| F5| FLNA| FN1| FYN| LMAN1| LRP1| LYN| PROS1| PTEN| TFPI| VTN| YWHAZ	**+**	**+**
hsa-miR-23b	hsa-miR-23b-3p|hsa-miR-23b-5p	**+**	**+**	**+**	**+**	**4**	APP| CALM1| CAV1| CBL| FLNA| FN1| FYN| LMAN1| PTEN| SERPING1| YWHAZ	**+**	**+**
hsa-miR-374a	hsa-miR-374a-3p|hsa-miR-374a-5p	**+**	**+**	**+**	**+**	**4**	APP| CALM1| CAV1| CBL| FN1| FYN| LMAN1| LYN| PTEN| TFPI	**+**	**+**
hsa-miR-128	hsa-miR-128-3p	**+**	**+**	**+**	**+**	**4**	APP| CALM1| CAV1| COL1A1| FLNA| FN1| FYN| PROS1| PTEN| SERPING1| YWHAZ	-	**+**
hsa-miR-124	hsa-miR-124-3p	**+**	**+**	-	**+**	**3**	ALB| APP| CALM1| CAV1| CBL| COL1A1| F5| FGA| FGB| FLNA| FN1| FYN| LMAN1| LYN| PROS1| PTPN11| SERPING1| TFPI	-	**+**
hsa-miR-129-2	hsa-miR-129-2-3p	**+**	**+**	-	**+**	**3**	COL1A1| FLNA| FN1| LYN| PROS1| PTEN| PTPN11| SERPING1| TFPI| YWHAZ	-	**+**
hsa-let-7a	hsa-let-7a-3p|hsa-let-7a-5p	**+**	-	**+**	**+**	**3**	APP| CALM1| CAV1| CBL| COL1A1| FLNA| FN1| FYN| LRP1| LYN| TFPI| YWHAZ	**+**	**+**
hsa-miR-21	hsa-miR-21-3p|hsa-miR-21-5p	**+**	**+**	**+**	-	**3**	APP| CALM1| FGB| FN1| FYN| PROS1| PTEN| PTPN11| TFPI	**+**	**+**
hsa-miR-30a	hsa-miR-30a-3p|hsa-miR-30a-5p	-	**+**	**+**	**+**	**3**	ALB| APP| CALM1| CAV1| F2| FLNA| FN1| LMAN1| PTEN| PTPN11| VTN| YWHAZ	-	**+**
hsa-miR-146a	hsa-miR-146a-5p	**+**	**+**	-	-	**2**	CBL| LMAN1| PTPN11	**+**	**+**
hsa-miR-17	hsa-miR-17-3p|hsa-miR-17-5p	**+**	-	-	**+**	**2**	APP| CALM1| CAV1| CBL| FLNA| FN1| LRP1| LYN| PTEN| PTPN11| YWHAZ	-	-
hsa-miR-103a	hsa-miR-103a-3p	**+**	**+**	-	-	**2**	ALB| APP| CAV1| COL1A1| FGB| FLNA| PTEN	-	**+**
hsa-miR-107	hsa-miR-107	**+**	**+**	-	-	**2**	ALB| APP| CAV1| COL1A1| FGB| PTEN	-	**+**
hsa-miR-20a	hsa-miR-20a-3p|hsa-miR-20a-5p	**+**	**+**	-	-	**2**	APP| CALM1| CAV1| FLNA| FYN| LMAN1| LYN| PTEN| YWHAZ	-	-
hsa-miR-210	hsa-miR-210-3p	**+**	-	-	**+**	**2**	APP| CALM1| CAV1| F5| FN1| FYN| LMAN1| LYN| PROS1| PTEN| PTPN11| TFPI	-	-
hsa-miR-93	hsa-miR-93-3p|hsa-miR-93-5p	**+**	-	**+**	-	**2**	APP| CALM1| CAV1| FLNA| FYN| LMAN1| LRP1| PTEN| YWHAZ	**+**	**+**
hsa-let-7 g	hsa-let-7 g-3p|hsa-let-7 g-5p	-	-	**+**	**+**	**2**	APP| CALM1| CBL| COL1A1| FLNA| FN1| FYN| LRP1| LYN| YWHAZ	**+**	**+**
hsa-miR-147a	hsa-miR-147a	**+**	-	-	-	1	APP| F5| FLNA| FYN| LYN| PTEN| PTPN11| TFPI	**+**	**+**
hsa-miR-182	hsa-miR-182-5p	**+**	-	-	-	1	CALM1| CBL| COL1A1| F2| FYN| PROS1| PTEN| YWHAZ	-	**+**
hsa-miR-191	hsa-miR-191-3p|hsa-miR-191-5p	**+**	-	-	-	1	CBL| FLNA| FYN| LMAN1| PROS1| PTPN11| YWHAZ	-	-
hsa-miR-7	hsa-miR-7-5p	**+**	-	-	-	1	CALM1| CAV1| CBL| FLNA| FN1| PTPN11| TFPI	-	**+**
hsa-miR-26a	hsa-miR-26a-5p	**+**	-	-	-	1	APP| CALM1| CBL| FN1| LMAN1| PTEN| PTPN11| YWHAZ	-	-
hsa-miR-101	hsa-miR-101-3p|hsa-miR-101-5p	**+**	-	-	-	1	APP| COL1A1| FLNA| FYN| LMAN1| LYN| YWHAZ	-	-
hsa-miR-335	hsa-miR-335-3p|hsa-miR-335-5p	-	**+**	-	-	1	FN1| LRP1| PLG| PROS1| PTPN11| SERPING1| TFPI| VTN| YWHAZ	-	**+**
hsa-miR-181a	hsa-miR-181a-3p|hsa-miR-181a-5p	-	-	**+**	-	1	APP| CALM1| COL1A1| LMAN1| PTEN| PTPN11| TFPI	**+**	**+**
hsa-miR-221	hsa-miR-221-3p|hsa-miR-221-5p	-	-	**+**	-	1	APP| FLNA| LRP1| LYN| PTEN| PTPN11| YWHAZ	**+**	-
hsa-miR-9	hsa-miR-9-3p|hsa-miR-9-5p	-	-	**+**	-	1	CALM1| CAV1| COL1A1| F2| FYN| LRP1| PTEN	**+**	**+**
hsa-miR-19a	hsa-miR-19a-3p|hsa-miR-19a-5p	-	-	**+**	-	1	APP| CALM1| CAV1| FN1| FYN| PTEN	**+**	-
hsa-miR-130a	hsa-miR-130a-3p|hsa-miR-130a-5p	-	-	**+**	-	1	APP| COL1A1| FN1| PTEN	**+**	**+**
hsa-miR-22	hsa-miR-22-3p|hsa-miR-22-5p	-	-	-	**+**	1	CALM1| CAV1| CBL| COL1A1| FLNA| FN1| FYN| PTEN| TFPI| YWHAZ	-	-

### 3.2. Participants

Patient characteristics are presented in Tables 2,3. Cardiovascular disease includes coronary artery disease, myocardial infarction and atrial fibrillation. We did not observe any differences between healthy individuals and COVID-19 patients with regard to basic demographic data including sex, age and body mass index (BMI) (p = 0.254, p = 0.707, p = 0.421, respectively). Among the 79 patients included, 9 (11.4%) patients, who were admitted to intensive care unit (ICU), died within the median of 11 days.

**Table 2.** Participant’s characteristics.

Characteristics	COVID-19 patients (N = 79)	Healthy individuals (N = 32)	*P*-value
Sex (male) (%)	44 (55.7%)	14 (44%)	0.254
Age (years)	59.7 ± 14.6	58.47 ± 16.59	0.707
BMI	29.7 ± 6.58	28.6 ± 6.3	0.421
Hypertension (%)	35 (44.3%)	16 (50%)	0.585
DM (%)	17 (21.5%)	6 (17%)	0.525
Current smoking (%)	5 (6.3%)	4 (10%)	0.543
Asthma/COPD (%)	6 (7.6%)	3 (6.3%)	0.756
CVD (CAD, MI, AF) (%)	14 (17.7%)	4 (12.5%)	0.499

Data are presented as number and percentage or mean and standard deviation. Abbreviations: AF, Atrial fibrillation; BMI, body mass index; CAD, coronary artery disease; COPD, chronic obstructive pulmonary disease; CVD, cardiovascular disease; DM, diabetes mellitus; MI, myocardial infarction

**Table 3.** Patients characteristics.

Laboratory parameters	At admission (N = 79)	Day-7 (N = 71)	Day-21 (N = 18)	*P*-value
hsCRP (mg/dL)	5.3 [3.13–7.69]	1.87 [0.61–5.27]	0.5 [0.1–4.89]	<0.001
D-dimer (μg/mL)	1.15 [0.45–2.16]	0.80 [0.46–1.47]	0.72 [0.36–1.78]	0.654
PCT (ng/mL)	0.11 [0.07–0.14]	0.07 [0.04–0.09]	0.07 [0.04–0.13]	0.538
WBC (mg/dL)	6.49 ± 2.87	8.69 ± 2.81	6.98 ± 2.74	0.028
Neutrophils	4.58 ± 3.17	6.24 ± 2.75	4.23 ± 2.0	0.091
Lymphocyte	0.72 ± 0.28	1.46 ± 0.85	1.74 ± 0.97	0.015
Blood iron (μg/dL)	36.5 [26.25–59.25]	86 [72.75–106.75]	88.5 [51.25–115.5]	0.001
Ferritine (ng/mL)	633 ± 306.20	771 ± 569.76	855 ± 310.23	0.093

Data are presented as mean ± standard deviation (SD) and median and interquartile range based on the data distribution. P-value is calculated based on One-way ANOVA test for three groups comparison. ***p*** values marked with bold indicate statistically significant differences between the multiple groups <0.05. Abbreviations: BMI, body mass index; COPD, chronic obstructive pulmonary disease; hsCRP, high-sensitivity C-reactive protein; CVD, cardiovascular disease; DM, diabetes mellitus; PCT, procalcitonin; SD, standard deviation; WBC, white blood cells.

Patients’ laboratory data at 3 different time-points are presented in Table 3. There was a significant difference in WBC count (p = 0.028), increase in lymphocyte count and iron levels (p = 0.015, p = 0.001 respectively), and a decrease in CRP levels (p < 0.001) over time.

### 3.3. Alteration of circulating thrombosis-related miRNAs expressions

**Figure 4.**
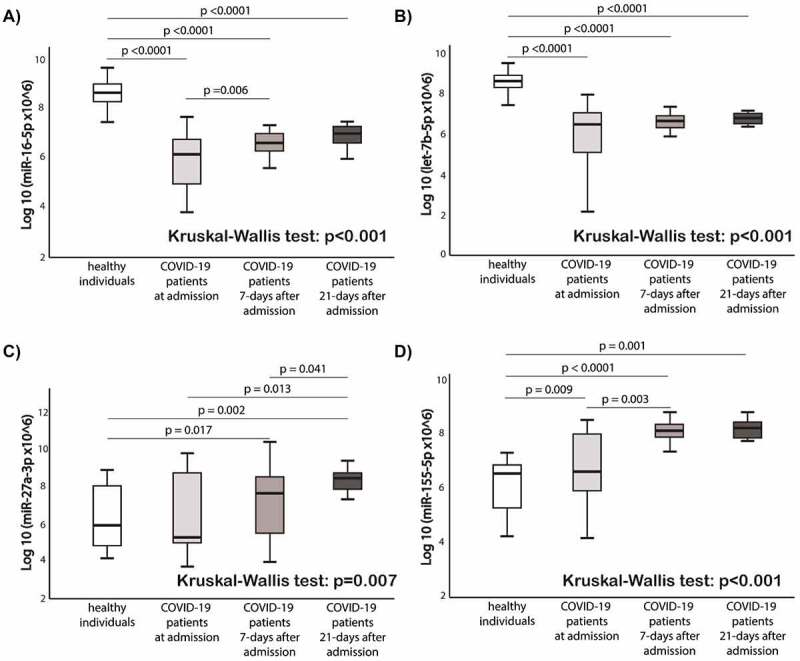
**Comparison of circulating miRNAs relative expression between the groups**. a) miR-16-5p; b) Let-7b-5p; c) miR-27a-3p; d) miR-155-5p. Mann-Whitney U test and Wilcoxon test were used appropriately. Kruskal-Wallis test shows the difference among the four groups. MiRNAs expression data is presented as log10 transformation. Abbreviations: COVID-19, coronavirus disease 2019; miR, microRNA; p, p value.

Fig. 4 shows the comparison of circulating miRNAs relative expression between healthy individuals and COVID-19 patients at 3 different timepoints (at admission, 7-days after admission, and 21-days after admission). The expression levels of miR-16-5p and let-7b in COVID-19 patients were lower at baseline, 7-days and 21-day after admission compared to the healthy controls (p < 0.0001 for all time points for both miRNAs). The expression levels of miR-27a-3p and miR-155-5p in COVID-19 patients were higher at day 21 compared to the healthy controls (p = 0.007 and p < 0.001, respectively). In COVID-19 patients, miR-27a-3p and miR-155-5p expressions increased over time. MiR-155-5p expression levels differed between healthy individuals and COVID-19 patients (p = 0.009).

### 3.4. Low baseline expression of miR-16-5p in patients with COVID-19 is associated with an increased hospital length of stay

**Figure 5.**
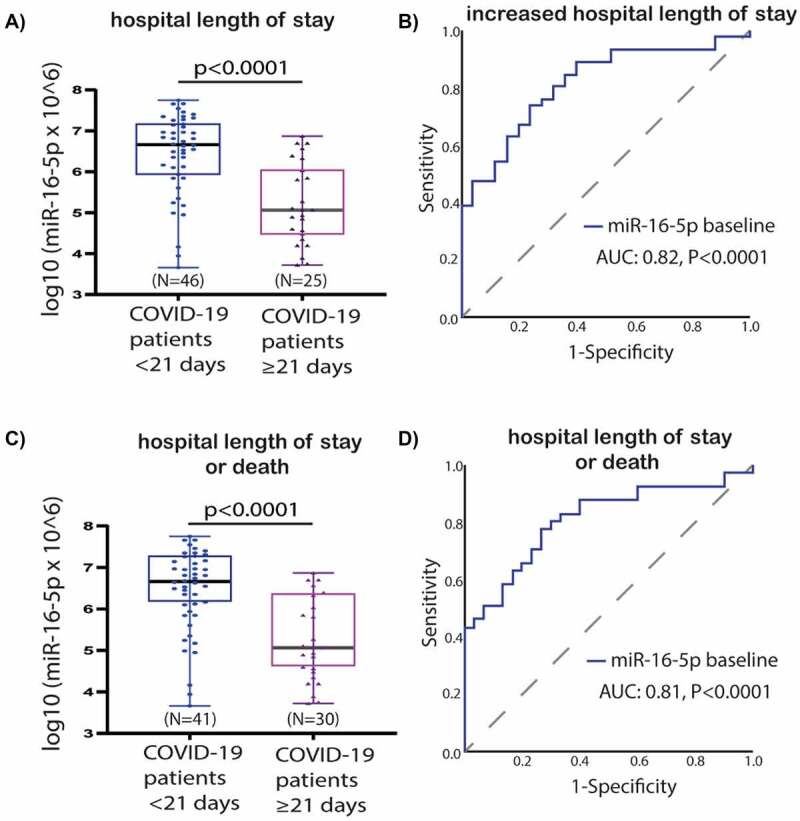
**Baseline miR-16-5p expression box-plots and receiver operating characteristic (ROC) curves**: a) miR-16-5p box-plots for hospital length of stay comparison; b) miR-16-5p ROC curve for prediction of hospital length of stay; c) miR-16-5p box-plots for hospital length of stay or death in follow-up as a composite endpoint; d) miR-16-5p ROC curve for prediction of hospital length of stay or death in follow-up as a composite endpoint. Abbreviations: AUC, Area under the ROC Curve; COVID-19, coronavirus disease 2019; miR, microRNA; N, number.

Patients hospitalized over a duration of 21 days had significantly lower expression levels of miR-16-5p when compared to those hospitalized under 21 days (p < 0.0001) (Fig. 5A,C). According to the ROC curve analysis, a low baseline miR-16-5p expression presents predictive utility in assessment of the hospital length of stay (AUC:0.815, p < 0.0001) (Fig. 5B). Similar results were found for the comparison of hospital length of stay or death in follow-up as a composite endpoint (AUC:0.810, 95% CI, 0.71–0.91, p < 0.0001) (Fig. 5D). No significant findings were observed according to other miRNAs whose expression levels were measured in our cohort (miR-27a-3p, let-7b-5p and miR-155-5p) **(Supplementary Figure 2)**.

The COVID-19 patients group was divided into two subgroups by using ROC curve analysis (based on hospital length of stay >21 days and/or death) for miR-16-5p, i.e. low- or high value (Fig. 5 **and** Table 4). The cut-off value of ≤ 6.1 was labelled as low miR-16-5p level (39% of the population). The sensitivity and specificity was 78% and 73% respectively for the cut off value. Similar results were found for ROC analysis based on a single endpoint of length of hospital stay > 21 days **(Supplementary Table 1)**. All the other analysed miRNAs (let-7b-5p, miR-27a-3p, miR-155-5p) did not present statistically significant predictive utility for increased hospitalization based on ROC curve analysis (data not shown).

**Table 4.** Statistical estimates for prediction of increased hospital length of stay or death in follow-up as a composite endpoint by baseline expression of miR-16-5p.

MiRNA	AUC (95% CI)	p-value	Cut-off	Sensitivity	Specificity	PPV	NPV
baseline low miR-16-5p vs high miR-16-5p	0.810 (0.71–0.91)	0.000009	6.1*	78%	73%	71%	80%

*Log10-transformed data was used for the cut-off value. Abbreviations: AUC, area under the curve; CI, confidence interval; PPV, positive predictive value; NPV, negative predictive value.

According to the multivariate logistic regression model, a low baseline miR-16-5p expression, together with diabetes mellitus (DM), were independent predictors of hospital length of stay >21 days or death (OR: 9.417; 95% CI, 2.647–33.506; p = 0.0005 and OR: 6.257; 95% CI, 1.049–37.316; p = 0.044, respectively) (Table 5). Similarly, a high baseline miR-16-5p expression was the only independent predictor of hospitalization time > 21 days (OR: 7.728; 95% CI, 2.253–26.508; p = 0.0012) **(Supplementary Table 2)**. Patients with high miR-16-5p expression levels at admission had approximately 9-fold and patients with DM 6-fold higher risk of longer hospitalization or death (Table 5).

**Table 5.** Multivariate logistic regression model for prediction of increased hospital length of stay or death in follow-up as a composite endpoint by low expression baseline of miR-16-5p along with clinical variables.

Variable	OR	95% CI		p-value
		Lower	Upper	
**Low baseline miR-16-5p expression**	9.417	2.647	33.506	**0.0005**
Gender (male)	1.580	0.373	6.686	0.534
Age (years)	1.034	0.970	1.103	0.303
BMI >30	1.449	0.379	5.535	0.588
**DM**	6.257	1.049	37.316	**0.044**
Smoking	1.934	0.101	37.062	0.661
Hypertension	0.677	0.134	3.421	0.637
CVD	0.245	0.037	1.616	0.144

*P* values marked with bold indicate statistically significant significant <0.05. Abbreviations: BMI, Body mass index; CI, confidence interval; COVID-19, coronavirus disease 2019; CVD, cardiovascular disease; DM, diabetes mellitus; miR, microRNA; OR, odds ratio.

## 4. Discussion

The aim of our study was to elucidate the expression pattern of circulating miRNAs associated with COVID-19 related thrombosis based on a bioinformatic analysis. We also compared the expressions of selected miRNAs between COVID-19 patients and healthy volunteers and monitored circulating miRNA expression patterns during the acute phase of COVID-19 disease, as well as the prognostic potential of these miRNAs as biomarkers. Among the possible strategies to select miRNAs for validation studies, we performed *in silico* bioinformatic analysis incorporating network medicine approaches. Such an approach allows us to summarize all available evidence regarding analysed biological processes and genes to generate predictions of specific targets and their molecular interactions.

The main findings of our study are: (i) based on *in silico* bioinformatic analysis the top miRNAs targeting thrombosis-related DE genes in response to SARS-CoV-2 infection were miR-16-5p, miR-27a-3p, let-7b-5p and miR-155-5p; (ii) the expression of miR-16-5p, miR-27a-3p and miR-155-5p increased during observation, compared to the baseline measurement; (iii) early expression changes were observed only for miR-155-5p, let-7b-5p and miR-16-5p when comparing to healthy controls; (iv) lower baseline expression of miR-16-5p and DM were independent predictors of increased length of stay or death according to a multivariate analysis.

### 4.1. Identification of the top genes related to thrombotic events associated with COVID-19

Analysis of ACE2 and coagulation interaction networks using NERI algorithm in four coronavirus infection-related expression datasets obtained from GEO database discovered multiple hub genes with corrupted signalling which can be responsible for thrombosis in COVID-19 patients. The most affected genes were EGFR, APP, HSP90AA1, TP53, PTEN, UBC, FN1, ELAVL1 and CALM1 (Figs. 2,3). Among the top genes playing a role in coagulation-related interaction networks in COVID-19, we identified CALM1 and enhancement of its connection with PTEN and UBC (Fig. 3). As we showed before, CALM1, APP, EGFR and ELAVL1 are crucial components of the ACE2 interaction network affected by SARS-Cov2 infection in pluripotent stem cell-derived cardiomyocytes (hiPSC-CMs) [1].

**EGFR** production has been linked to thrombosis risk and inflammatory markers, and it was previously shown that viral infections may induce EGFR signalling and promote a pro-inflammatory and pro-angiogenic response [63,64]. Overactive EGFR signalling also leads to increased fibrosis after SARS coronavirus infection [65]. **APP** (amyloid-β precursor protein gene), is a gene encoding APP protein, a precursor for amyloid beta (Aβ) peptide. Platelets, a main source of Aβ in circulation, can be activated via various factors, i.e. inflammation and viral antigens. Our recent bioinformatic analysis pointed to APP and PTEN as the top genes involved in platelet activity and most susceptible for noncoding regulation by platelet-related miRNAs [66]. Upon their activation, platelets release APP and Aβ into the circulation. Moreover, APP is cleaved by endothelial cells, creating Aβ. Aβ promotes the activation of the coagulation factor XII, which results in stimulation of thrombin generation, possibly creating a prothrombotic state [67,68]. It was shown that Aβ may play a role not only in haemostasis but also in inflammation [67,69,70]. This makes it an important finding for elucidating the COVID-19-related thrombosis but also potentially COVID-19 related neurodegeneration due to its association with Alzheimer’s disease. Another DE gene from our network is **HSP90AA1**, which codes heat shock protein 90α (Hsp90α), inducing inflammation through activation of the NF-kB and STAT3 pathways, while NF-kB also induces the expression of Hsp90α [71]. In human cell lines infected with SARS-CoV and SARS-CoV-2, HSP90 inhibition resulted in reduction of viral replication, which suggests its involvement in infection []. Taking into account recent studies pointing out HSP90 as a potential target in COVID-19 treatment [72,73,74,75], this finding especially supports the value of presented here results.

Another top gene identified in this study was **ELAVL1**, which encodes HuR protein, playing a critical role in post-transcriptional regulation, splicing and suppression of thrombomodulin synthesis in interleukin-1β (IL-1β) treatment and sepsis [76]. Thrombomodulin is a critical factor to activate protein C (aPC) in mediating the anticoagulation and anti-inflammation effects. Additionally, a recent study showed that there is a significant positive correlation between ELAVL1 and ACE2 in chronic obstructive pulmonary disease (COPD) cells [77]. This result especially points out the direct link between thrombosis and SARS-Cov-2 receptor ACE.

**CALM1**, which encodes a highly conservative protein, a key calcium sensor – calmodulin (CaM), represents another interesting candidate in our study. CaM interacts with viral proteins and positively influences the propagation of rotavirus infection [78]. CaM also regulates the activation of GPVI and GPIb-IX-V platelet receptors and NOS activation, which means that it has influence on platelet and endothelial homoeostasis [79,80]. Moreover, CALM1 is also an important ACE2 interactor, and is playing a role in viral pathogenesis [77,81,82]. In our analysis we observed alteration by coronaviruses and its connection with PTEN, EGFR, FLNA, ELAVL1 and NXF involved in regulation of the platelet activity and inflammatory processes. This suggests the important role of CALM1 in COVID-19 related thrombotic complications. Moreover, in enrichment analyses we found strong overrepresentation of pathways related to immune system and platelet activity pointing out that genes identified based on publicly available expression data indeed play a role in thrombosis processes induced by coronavirus infection.

### 4.2. Identification of the top miRNAs related to thrombotic events associated with COVID-19

The identification of the top genes related to thrombosis and ACE2 interaction network, affected by coronavirus infection, allowed us to predict the miRNAs with the biggest potential of playing a role as biomarkers in thrombotic complications associated with COVID-19. **MiR-16-5p** takes part in the regulation of inflammation and programmed cell death. Many studies showed the anti-inflammatory effect of miR-16 in atherosclerosis, acute lung injury and sepsis [83,84,85]. MiR-16 influences the phenotypic changes on T cells survival, differentiation, and proliferation, which are critical for antiviral responses. Limited studies aimed to analyse the predictive and prognostic value of miR-16-5p, and miR-27a in cardiovascular diseases. A previous publication showed up-regulation of miR-16 in patients with Takotsubo cardiomyopathy compared to healthy subjects [86]. Latest meta analysis assessed the prognostic utility of 19 circulating miRNAs included in four relevant articles in heart failure patients. Meta analysis showed that patients with low expression of miR-16-5p and miR-27a levels have significantly worse overall survival [87]. MiR-27a was found significantly lower in patients with atherosclerosis compared to healthy individuals, however the study included only 25 patients and 26 controls [88]. However, its impact on SARS-CoV2 infection remains unclear. Previous bioinformatic analysis revealed the ability of miR-16-5p to target genes responsible for host-SARS-CoV2 interaction [1,61]. Another possible implication of this miRNA in COVID-19 infections is its ability to influence viral entry receptor (ACE2) related networks, which was also determined by bioinformatic analyses [1,89]. However, the implications of these findings have not been deeply investigated yet. One possible explanation is that miR-16 suppresses cell cycle and prevents viral replication through decreasing CCND1 level, which is a crucial protein in G1 to S phase transition during cell cycle processes [61,90].

In line with our data, miR-16-5p represents the most abundant miRNA in the plasma, followed by miR-223-3p, let-7b-5p and miR-146a-5p in previous study [91]. Further several miRNAs, including miR-16-2-3p, DE in peripheral blood and infected tissues from COVID-19 patients when compared to healthy controls, indicate a potential role in the infection [92,93]. However miR-16-5p is downregulated in lung epithelium, but upregulated in peripheral blood [92,93]. Importantly, in another study, plasma levels of miR-16-5p were lower in critically ill patients and were negatively correlated with the total number of days of ICU stay (rho = −0.38). Besides, miR-16-5p in cluster with miR-92a-3p, miR-98-5p, miR-132-3p, miR-192-5p and miR-323a-3p showed significant decrease in patients who did not survive at the ICU, however miR-16-5p was not included in the multivariate analysis that predicted mortality during the ICU stay [43]. Also in our analysis we did not find a correlation between miR-16-5p expression and mortality, although it was significantly lower in individuals who stayed longer in hospital.

Thrombosis-related **miR-27a-3p** also influences ACE2 related pathways [1] and its expression was upregulated in hospitalized COVID-19 patients compared to healthy controls. We also observed a significant increase in its expression during the stay in hospital, however there was no association with in-hospital length of stay or mortality. MiR-27a-3p is DE between ward and ICU patients, was correlated with leukocytes and CRP [43], and represents a potential in regulation of inflammatory response in sepsis. MiR-27a stimulates the synthesis of inflammatory cytokines through the NF-kB pathway, and thus IL-6 and TNF-a expression. Thus, it may be hypothesized that miR-27a promotes pulmonary inflammation and sepsis or may reflect SARS-CoV-2 mediated gastrointestinal tract infection or inflammation [94].

In a previous study which performed small RNA deep sequencing in patients with COVID-19, miR-16-5p was the most abundant regulated miRNA, followed by miR-223-3p, let-7b-5p and miR-146a-5p [91]. Moreover, previously published bioinformatic analysis also presented the interaction between **let-7b-5p** and TMPRSS [95]. Based on our bioinformatic prediction analysis we validated the potential of let-7b in COVID-19 patients and found that low expressions of let-7b can have diagnostic utility in patients with COVID-19. Let-7b can potentially enhance the synthesis of inflammatory cytokines, activating TLR4/NF-κB pathway and inhibiting the TLR4 expression, which in COVID-19 patients may be interesting especially in neutrophils, due to their role in sepsis development. Thus, let-7b might indirectly stimulate neutrophils recruitment [96]. Let-7b further inhibits M protein expression of the *SARS-CoV-2* genome. Interestingly, let-7 not only IL-6 mRNA expression, but also significantly decreased the expression of many other SARS-CoV-2 related cytokines and chemokines including IL-1β, IL-8, CCL2, GM-CSF, TNF-α, and VEGFα. Therefore, let-7, a miRNA ubiquitously expressed in human cells, blocks SARS-CoV-2 replication by targeting S and M protein, as well as inhibits the expression of multiple inflammatory mediators [97].

As previously described miRNAs, **miR-155** is also involved in inflammation. There it acts via different mechanisms by directly targeting IL13Rα1, IL-13 receptor and also contributes to IL-8 secretion. Moreover, it influences the T-cell differentiation and affects the innate immunity [72]. MiR-155 additionally regulates pathways related with the IFN superfamily, an important regulator of inflammatory response, especially in viral infections [98,99,100]. SARS-CoV-2 infection correlates with a strong increase of miR-155 expression in the infected cells and thereby allows the distinction between patients with influenza-related and COVID-19-related acute respiratory distress syndromes [101]. Recently two human studies found that miR-155 expression significantly differed between critically ill COVID-19 patients and healthy controls [102,103]. Importantly, Tacke *et al*. [103] did not find miR-155 as an indicator for patient’s survival. However, when patients were subdivided according to their age upon admission to the ICU into those younger and older than 65 years, low miR-155 expression levels became a strong independent indicator for patient mortality [103]. In our study only miR-155-5p expression was significantly higher in COVID-19 patients compared to healthy individuals. Moreover, miR-155 expression showed an increasing trend at day-7 and day-21 after admission. Our results are in line with previous reports which stated that high miR-155 expression can be a diagnostic biomarker. Further analysis should confirm its ability to predict the severity of the disease in COVID-19 patients.

## 5. Study limitations

The main limitation of our study is the small size of the patient group and a low mortality in the included cohort. Moreover, there were no clinically relevant thrombotic events in the patient population, and thus we could not test the hypothesis whether miRNAs selected based on computational analysis may serve as predictive biomarkers of thrombo-inflammatory events. Third, due to the hypothesis-generating study design, we limited our analysis to miRNAs associated with thrombosis, based on our bioinformatic analysis and publicly available expression datasets related to coronavirus infection. However, we did not perform the miRNA sequencing in the collected plasma samples, which might enable us to determine novel miRNAs with higher predictive value for COVID-19. Finally, we did not have the possibility to analyse the top genes that were predicted in relation to thrombosis from our bioinformatic analysis. Altogether, our results should be confirmed in a larger, preferably multi-centre study before miRNAs and their target genes can be used as a prognostic biomarker of COVID-19 in clinical practice.

## 6. Conclusions

This study enabled us to better characterize changes in gene expression and signalling pathways related to hypercoagulable and thrombotic conditions in COVID-19. In this study we identified and validated miRNAs which could serve as novel, thrombosis-related predictive biomarkers of the COVID-19 complications, and can be used for early stratification of patients and prediction of severity of infection development in an individual. Non-coding RNAs associated with inflammation and coagulation pathways identified by bioinformatic analysis can serve as potential early biomarkers helping in identification of the pathological changes in COVID-19. Future investigations should take into account the effect of pharmacological therapies on the circulating miRNAs pattern in this emerging disease [43]. Identifying novel biomarkers and creating predictive tools may improve outcome in patients with COVID-19, and therefore has a potential for reducing disease-related, personal and economic consequences of the global pandemic. Additional studies in larger cohorts with thrombotic complications and functional approaches are warranted to validate these findings and provide further insight into the role of circulating miRNAs as biomarkers and functional mediators of COVID-19.

## Supplementary Material

Supplemental Material

## Data Availability

The data that support the findings of this study are openly available in https://www.scidb.cn/s/J3aa6f. The data entitled ‘Eyileten et al. COVID-19 miRNA’ was uploaded by Eyileten C.

## References

[1] Wicik Z, Eyileten C, Jakubik D, et al. ACE2 interaction networks in COVID-19: a physiological framework for prediction of outcome in patients with cardiovascular risk factors. J Clin Med Res. 2020;9. DOI:10.3390/jcm9113743. PMC7700637 33233425

[2] Aziz F, Aberer F, Bräuer A, et al. COVID-19 in-hospital mortality in people with diabetes is driven by comorbidities and age—propensity score-matched analysis of Austrian national public health institute data. Viruses. 2021;13(12):2401. 34960670 10.3390/v13122401 PMC8705658

[3] Gupta A, Madhavan MV, Sehgal K, et al. Extrapulmonary manifestations of COVID-19. Nat Med. 2020;26(7):1017–1032. 32651579 10.1038/s41591-020-0968-3 PMC11972613

[4] Gu SX, Tyagi T, Jain K, et al. Thrombocytopathy and endotheliopathy: crucial contributors to COVID-19 thromboinflammation. Nat Rev Cardiol. 2021;18(3):194–209. 33214651 10.1038/s41569-020-00469-1 PMC7675396

[5] Jackson SP, Darbousset R, Schoenwaelder SM. Thromboinflammation: challenges of therapeutically targeting coagulation and other host defense mechanisms. Blood. 2019;133(9):906–918. 30642917 10.1182/blood-2018-11-882993

[6] Bikdeli B, Madhavan MV, Gupta A, et al. Pharmacological agents targeting thromboinflammation in COVID-19: Review and implications for future research. Thromb Haemost. 2020;120(7):1004–1024. 32473596 10.1055/s-0040-1713152 PMC7516364

[7] McGonagle D, O’Donnell JS, Sharif K, et al. Immune mechanisms of pulmonary intravascular coagulopathy in COVID-19 pneumonia. Lancet Rheumatol. 2020;2(7):e437–e445. 30121-1. 32835247 10.1016/S2665-9913(20)30121-1 PMC7252093

[8] Ackermann M, Verleden SE, Kuehnel M, et al. Pulmonary vascular endothelialitis, thrombosis, and angiogenesis in Covid-19. N Engl J Med. 2020;383(2):120–128. 32437596 10.1056/NEJMoa2015432 PMC7412750

[9] Gómez CA, Sun C-K, Tsai I-T, et al. Mortality and risk factors associated with pulmonary embolism in coronavirus disease 2019 patients: a systematic review and meta-analysis. Sci Rep. 2021;11(1):16025. 34362946 10.1038/s41598-021-95512-7 PMC8346591

[10] Bonaventura A, Vecchié A, Dagna L, et al. Endothelial dysfunction and immunothrombosis as key pathogenic mechanisms in COVID-19. Nat Rev Immunol. 2021;21(5):319–329. 33824483 10.1038/s41577-021-00536-9 PMC8023349

[11] Thachil J, Tang N, Gando S, et al. ISTH interim guidance on recognition and management of coagulopathy in COVID-19. J Thromb Haemost. 2020;18(5):1023–1026. 32338827 10.1111/jth.14810 PMC9906133

[12] Bikdeli B, Madhavan MV, Jimenez D, et al. COVID-19 and thrombotic or thromboembolic disease: implications for prevention, antithrombotic therapy, and follow-up: JACC State-of-the-art review. J Am Coll Cardiol. 2020;75(23):2950–2973. 32311448 10.1016/j.jacc.2020.04.031 PMC7164881

[13] Agyemang C, van den Born B-J, Gorog DA, Storey RF, Gurbel PA, Tantry US, Berger JS, Chan MY, et al. Current and novel biomarkers of thrombotic risk in COVID-19: a consensus statement from the international COVID-19 thrombosis biomarkers colloquium. Nat Rev Cardiol. 2022;19(1):1–21. 35027697 10.1038/s41569-021-00665-7 PMC8757397

[14] Friedman RC, Farh KK-H, Burge CB, et al. Most mammalian mRNAs are conserved targets of microRNAs. Genome Res. 2009;19(1):92–105. 18955434 10.1101/gr.082701.108 PMC2612969

[15] Pordzik J, Jakubik D, Jarosz-Popek J, et al. Significance of circulating microRNAs in diabetes mellitus type 2 and platelet reactivity: bioinformatic analysis and review. Cardiovasc Diabetol. 2019;18(1):113. 31470851 10.1186/s12933-019-0918-x PMC6716825

[16] Wang Y, Liu C, Wei W, et al. Predictive value of circulating coagulation related microRNAs expressions for major adverse cardiac and cerebral event risk in patients undergoing continuous ambulatory peritoneal dialysis: a cohort study. J Nephrol. 2020;33(1):157–165. 31359371 10.1007/s40620-019-00626-x PMC7007420

[17] Eyileten C, Wicik Z, De Rosa S, et al. MicroRNAs as diagnostic and prognostic biomarkers in ischemic stroke-A comprehensive review and bioinformatic analysis. Cells. 2018;7. DOI:10.3390/cells7120249. PMC6316722 30563269

[18] Pordzik J, Pisarz K, De Rosa S, et al. The potential role of platelet-related microRNAs in the development of cardiovascular events in high-risk populations, including diabetic patients: a review. Front Endocrinol. 2018;9:74. 10.3389/fendo.2018.00074 PMC5869202 29615970

[19] Sabatino J, Wicik Z, De Rosa S, et al. MicroRNAs fingerprint of bicuspid aortic valve. J Mol Cell Cardiol. 2019;134:98–106. 31278905 10.1016/j.yjmcc.2019.07.001

[20] Eyileten C, Sharif L, Wicik Z, et al. The relation of the brain-derived neurotrophic factor with microRNAs in neurodegenerative diseases and ischemic stroke. Mol Neurobiol. 2021;58(1):329–347. 32944919 10.1007/s12035-020-02101-2 PMC7695657

[21] Wolska M, Jarosz-Popek J, Junger E, et al. Long non-coding RNAs as promising therapeutic approach in ischemic stroke: a comprehensive review. Mol Neurobiol. 2020;58(4):1664–1682. 33236327 10.1007/s12035-020-02206-8 PMC7932985

[22] Soplinska A, Zareba L, Wicik Z, et al. MicroRNAs as biomarkers of systemic changes in response to endurance exercise-A comprehensive review. Diagnostics (Basel). 2020;10. DOI:10.3390/diagnostics10100813. PMC7602033 33066215

[23] Zareba L, Fitas A, Wolska M, et al. MicroRNAs and long noncoding RNAs in coronary artery disease: new and potential therapeutic targets. Cardiol Clin. 2020;38(4):601–617. 33036721 10.1016/j.ccl.2020.07.005

[24] Gasecka A, Siwik D, Gajewska M, et al. Early biomarkers of neurodegenerative and neurovascular disorders in diabetes. J Clin Med Res. 2020;9. DOI:10.3390/jcm9092807. PMC7564566 32872672

[25] Jarosz-Popek J, Wolska M, Gasecka A, et al. The importance of non-coding RNAs in neurodegenerative processes of diabetes-related molecular pathways. J Clin Med Res. 2020;10. DOI:10.3390/jcm10010009. PMC7793080 33374507

[26] Morelli VM, Brækkan SK, Hansen J-B. Role of microRNAs in venous thromboembolism. Int J Mol Sci. 2020;21. DOI:10.3390/ijms21072602 PMC7177540 32283653

[27] Kim AS, Kalady MF, DeVecchio J, et al. Identifying miRNA biomarkers and predicted targets associated with venous thromboembolism in colorectal cancer patients. Blood. 2019;134(Supplement_1):3643.

[28] Xiao J, Jing Z-C, Ellinor PT, et al. MicroRNA-134 as a potential plasma biomarker for the diagnosis of acute pulmonary embolism. J Transl Med. 2011;9(1). DOI:10.1186/1479-5876-9-159 PMC3189884 21943159

[29] Kessler T, Erdmann J, Vilne B, et al. Serum microRNA-1233 is a specific biomarker for diagnosing acute pulmonary embolism. J Transl Med. 2016;14(1). DOI:10.1186/s12967-016-0886-9 PMC4858885 27150028

[30] Zhou X, Wen W, Shan X, et al. MiR-28-3p as a potential plasma marker in diagnosis of pulmonary embolism. Thromb Res. 2016;138:91–95. 26702486 10.1016/j.thromres.2015.12.006

[31] Qin J, Liang H, Shi D, et al. A panel of microRNAs as a new biomarkers for the detection of deep vein thrombosis. J Thromb Thrombolysis. 2015;39(2):215–221. 25244974 10.1007/s11239-014-1131-0

[32] Ronco C, Bellomo R, and Kellum JA, et al. Critical care nephrology e-book. Elsevier health sciences; 2017 [cited 2022 July 18]. Available from: https://play.google.com/store/books/details?id=HTdDDwAAQBAJ

[33] Jiang Z, Ma J, Wang Q, et al. Combination of circulating miRNA-320a/b and D-dimer improves diagnostic accuracy in deep vein thrombosis patients. Med Sci Monit. 2018;24:2031–2037. 29622762 10.12659/MSM.906596 PMC5903311

[34] Brown JAL, Bourke E. Practical bioinformatics analysis of MiRNA data using online tools. Methods Mol Biol. 2017;1509:195–208. 27826929 10.1007/978-1-4939-6524-3_18

[35] A-L B, Gulbahce N, Loscalzo J. Network medicine: a network-based approach to human disease. Nat Rev Genet. 2011;12(1):56–68. 21164525 10.1038/nrg2918 PMC3140052

[36] Simões SN, Martins DC, Pereira CAB, et al. NERI: network-medicine based integrative approach for disease gene prioritization by relative importance. BMC Bioinformatics. 2015;16(S19):1–14. 26696568 10.1186/1471-2105-16-S19-S9 PMC4686785

[37] Chang X, Xu T, Li Y, et al. Dynamic modular architecture of protein-protein interaction networks beyond the dichotomy of “date” and “party” hubs. Sci Rep. 2013;3(1):1–8. 10.1038/srep01691 PMC3631766 23603706

[38] Agarwal S, Deane CM, Porter MA, Agarwal S, Deane CM, Porter MA, Jones NS. Revisiting Date and. Party hubs: novel approaches to role assignment in protein interaction networks. PLoS Comput Biol. 2010;6(6):e1000817. 20585543 10.1371/journal.pcbi.1000817 PMC2887459

[39] Schulte C, Barwari T, Joshi A, et al. Comparative analysis of circulating noncoding RNAs versus protein biomarkers in the detection of myocardial injury. Circ Res. 2019;125(3):328–340. 31159652 10.1161/CIRCRESAHA.119.314937 PMC6641471

[40] Eyileten C, Jakubik D, Shahzadi A, et al. Diagnostic performance of circulating miRNAs and extracellular vesicles in acute ischemic stroke. Int J Mol Sci. 2022;23. DOI:10.3390/ijms23094530. PMC9102701 35562921

[41] Messner CB, Demichev V, Wendisch D, et al. Ultra-high-throughput clinical proteomics reveals classifiers of COVID-19 infection. Cell Syst. 2020;11(1):11–24.e4. 32619549 10.1016/j.cels.2020.05.012 PMC7264033

[42] Gutmann C, Takov K, Burnap SA, et al. SARS-CoV-2 RNAemia and proteomic trajectories inform prognostication in COVID-19 patients admitted to intensive care. Nat Commun. 2021;12(1):3406. 34099652 10.1038/s41467-021-23494-1 PMC8184784

[43] de Gonzalo-Calvo D, Benítez ID, Pinilla L, Gonzalo-Calvo D de, Benítez ID, Pinilla L, Carratalá A, Moncusí-Moix A, Gort-Paniello C, et al. Circulating microRNA profiles predict the severity of COVID-19. in hospitalized patients. Transl Res. 2021;236:147–159. 34048985 10.1016/j.trsl.2021.05.004 PMC8149473

[44] Badimon L, Robinson EL, Jusic A, et al. Cardiovascular RNA markers and artificial intelligence may improve COVID-19 outcome: a position paper from the EU-CardioRNA COST Action CA17129. Cardiovasc Res. 2021;117(8):1823–1840. 33839767 10.1093/cvr/cvab094 PMC8083253

[45] Gutmann C, Khamina K, Theofilatos K, et al. Association of cardiometabolic microRNAs with COVID-19 severity and mortality. Cardiovasc Res. 2022;118(2):461–474. 34755842 10.1093/cvr/cvab338 PMC8689968

[46] Palasca O, Santos A, Stolte C, et al. 2.0: an integrative web resource on mammalian tissue expression. Database. 2018;2018. DOI:10.1093/database/bay003. PMC5808782 29617745

[47] Shannon P, Markiel A, Ozier O, et al. Cytoscape: a software environment for integrated models of biomolecular interaction networks. Genome Res. 2003;13(11):2498–2504. 14597658 10.1101/gr.1239303 PMC403769

[48] Doncheva NT, Morris JH, Gorodkin J, et al. Cytoscape stringApp: network analysis and visualization of proteomics data. J Proteome Res. 2019;18(2):623–632. 30450911 10.1021/acs.jproteome.8b00702 PMC6800166

[49] Reghunathan R, Jayapal M, Hsu L-Y, Chng H-H, Tai D, Leung BP, et al. BMC Immunology. p. 2. 2005. DOI:10.1186/1471-2172-6-2 PMC546205 15655079

[50] Sharma A, Garcia G, Wang Y, Plummer J T, Morizono K, Arumugaswami V, Svendsen C N. (2020). Human iPSC-Derived Cardiomyocytes Are Susceptible to SARS-CoV-2 Infection. Cell Rep Med, 1(4), 100052 10.1016/j.xcrm.2020.100052 32835305 PMC7323681

[51] Desai N *et al* . (2020). Temporal and spatial heterogeneity of host response to SARS-CoV-2 pulmonary infection. Nat Commun, 11(1), 6319 10.1038/s41467-020-20139-7 33298930 PMC7725958

[52] Wicik Z, Jales Neto LH, Guzman LEF, et al. The crosstalk between bone metabolism, lncRNAs, microRNAs and mRNAs in coronary artery calcification. Genomics. 2021;113(1):503–513. 32971215 10.1016/j.ygeno.2020.09.041

[53] Ru Y, Kechris KJ, Tabakoff B, et al. The multiMiR R package and database: integration of microRNA–target interactions along with their disease and drug associations. Nucleic Acids Res. 2014;42(17):e133. 25063298 10.1093/nar/gku631 PMC4176155

[54] Chen E Y, Tan C M, Kou Y, Duan Q, Wang Z, Meirelles G Vaz, Clark N R, Ma'ayan A. (2013). Enrichr: interactive and collaborative HTML5 gene list enrichment analysis tool. BMC Bioinformatics, 14 128 10.1186/1471-2105-14-128 23586463 PMC3637064

[55] wma) WMA, World Medical Association (WMA). Declaration of Helsinki. Ethical Principles for Medical Research Involving Human Subjects. Jahrbuch für Wissenschaft und Ethik. pp. 233–238. 2009. DOI:10.1515/9783110208856.233

[56] De Rosa S, Eposito F, Carella C, et al. Transcoronary concentration gradients of circulating microRNAs in heart failure. Eur J Heart Fail. 2018;20(6):1000–1010. 29314582 10.1002/ejhf.1119

[57] De Rosa R, De Rosa S, Leistner D, et al. Transcoronary concentration gradient of microRNA-133a and outcome in patients with coronary artery disease. Am J Cardiol. 2017;120(1):15–24. 28511772 10.1016/j.amjcard.2017.03.264

[58] Eyileten C, Fitas A, Jakubik D, et al. Alterations in circulating MicroRNAs and the relation of MicroRNAs to maximal oxygen consumption and intima–media thickness in ultra-marathon runners. Int J Environ Res Public Health. 2021;18(14):7234. 34299680 10.3390/ijerph18147234 PMC8307599

[59] Pordzik J, Eyileten-Postuła C, Jakubik D, et al. MiR-126 is an independent predictor of long-term all-cause mortality in patients with type 2 diabetes mellitus. J Clin Med Res. 2021;10. DOI:10.3390/jcm10112371. PMC8198825 34071189

[60] Eyileten C, Wicik Z, Keshwani D, et al. Alteration of circulating platelet-related and diabetes-related microRNAs in individuals with type 2 diabetes mellitus: a stepwise hypoglycaemic clamp study. Cardiovasc Diabetol. 2022;21(1):79. 35596173 10.1186/s12933-022-01517-5 PMC9123651

[61] Jafarinejad-Farsangi S, Jazi MM, Rostamzadeh F, et al. High affinity of host human microRNAs to SARS-CoV-2 genome: an in silico analysis. Noncoding RNA Res. 2020;5(4):222–231. 33251388 10.1016/j.ncrna.2020.11.005 PMC7680021

[62] Ahmadi A, Moradi S. In silico analysis suggests the RNAi-enhancing antibiotic enoxacin as a potential inhibitor of SARS-CoV-2 infection. Sci Rep. 2021;11(1):10271. 33986351 10.1038/s41598-021-89605-6 PMC8119475

[63] Hamdy NM, El-Wakeel L, Suwailem SM. Involvement of depressive catecholamines as thrombosis risk/inflammatory markers in non-smoker, non-obese congestive heart failure, linked to increased epidermal growth factor-receptor (EGF-R) production. Indian J Clin Biochem. 2011;26(2):140–145. 22468040 10.1007/s12291-010-0106-y PMC3107402

[64] Mitchell HD, Eisfeld AJ, Stratton KG, et al. The role of EGFR in influenza pathogenicity: multiple network-based approaches to identify a key regulator of non-lethal infections. Front Cell Dev Biol. 2019;7:200. 31616667 10.3389/fcell.2019.00200 PMC6763731

[65] Venkataraman T, Coleman CM, Frieman MB. Overactive epidermal growth factor receptor signaling leads to increased fibrosis after severe acute respiratory syndrome coronavirus infection. J Virol. 2017;91(12). DOI:10.1128/jvi.00182-17 PMC5446658 28404843

[66] Wicik Z, Czajka P, Eyileten C, et al. The role of miRNAs in regulation of platelet activity and related diseases - a bioinformatic analysis. Platelets. 2022 [citied 2022 Jun 11];1–13. DOI:10.1080/09537104.2022.2042233 35285386

[67] Inyushin M, Zayas-Santiago A, Rojas L, et al. Platelet-generated amyloid beta peptides in Alzheimer’s disease and glaucoma. Histol Histopathol. 2019;34(8):843–856. 30945258 10.14670/HH-18-111 PMC6667289

[68] Zamolodchikov D, Renné T, The Alzheimer’s SS. disease peptide β-amyloid promotes thrombin generation through activation of coagulation factor XII. J Thromb Haemost. 2016;14(5):995–1007. 26613657 10.1111/jth.13209 PMC4870142

[69] Kam T-I, Song S, Gwon Y, et al. FcγRIIb mediates amyloid-β neurotoxicity and memory impairment in Alzheimer’s disease. J Clin Invest. 2013;123(7):2791–2802. 23921129 10.1172/JCI66827 PMC3696552

[70] Ahn HJ, Chen Z-L, Zamolodchikov D, et al. Interactions of β-amyloid peptide with fibrinogen and coagulation factor XII may contribute to Alzheimer’s disease. Curr Opin Hematol. 2017;24(5):427–431. 28661939 10.1097/MOH.0000000000000368 PMC5540762

[71] Zuehlke AD, Beebe K, Neckers L, et al. Regulation and function of the human HSP90AA1 gene. Gene. 2015;570(1):8–16. 26071189 10.1016/j.gene.2015.06.018 PMC4519370

[72] Wyler E, Mösbauer K, Franke V, et al. Transcriptomic profiling of SARS-CoV-2 infected human cell lines identifies HSP90 as target for COVID-19 therapy. iScience. 2021;24(3):102151. 33585804 10.1016/j.isci.2021.102151 PMC7866843

[73] Ramos CHI, Ayinde KS. Are Hsp90 inhibitors good candidates against Covid-19? Curr Protein Pept Sci. 2020. cited 2022 Jun 11. DOI:10.2174/1389203721666201111160925. 33176644

[74] Li C, Chu H, Liu X, et al. Human coronavirus dependency on host heat shock protein 90 reveals an antiviral target. Emerg Microbes Infect. 2020;9(1):2663–2672. 33179566 10.1080/22221751.2020.1850183 PMC7751432

[75] Goswami R, Russell VS, and Tu JJ, et al. Oral Hsp90 inhibitor, SNX-5422, attenuates SARS-CoV-2 replication and dampens inflammation in airway cells. iScience. 24(12) 103412. DOI:10.1016/j.isci.2021.103412. PMC8579697 34786537

[76] Lan C, Shi X, Guo N, et al. [Value of serum miR-155-5p and miR-133a-3p expression for the diagnosis and prognosis evaluation of sepsis]. Zhonghua Wei Zhong Bing Ji Jiu Yi Xue. 2016;28(8):694–698. 27434558 10.3760/cma.j.issn.2095-4352.2016.08.005

[77] Hori K, Shimaoka K, Hoshino M, Aloufi N, Haidar Z, Ding J, Nair P, Benedetti A, Eidelman DH, et al. Role of human antigen r (hur) in the regulation of pulmonary ACE2 expression. Cells. 2021;11(1):11. 35011584 10.3390/cells11010022 PMC8750694

[78] Chattopadhyay S, Basak T, Nayak MK, et al. Identification of cellular calcium binding protein calmodulin as a regulator of rotavirus A infection during comparative proteomic study. PLoS One. 2013;8(2):e56655. 23437200 10.1371/journal.pone.0056655 PMC3577757

[79] Gardiner EE, Arthur JF, Berndt MC, et al. Role of calmodulin in platelet receptor function. Curr Med Chem Cardiovasc Hematol Agents. 2005;3(4):283–287. 16250859 10.2174/156801605774322283

[80] Tousoulis D, Kampoli A-M, Tentolouris C, et al. The role of nitric oxide on endothelial function. Curr Vasc Pharmacol. 2012;10(1):4–18. 22112350 10.2174/157016112798829760

[81] Lambert DW, Clarke NE, Hooper NM, et al. Calmodulin interacts with angiotensin-converting enzyme-2 (ACE2) and inhibits shedding of its ectodomain. FEBS Lett. 2008;582(2):385–390. 18070603 10.1016/j.febslet.2007.11.085 PMC7094239

[82] Perrin-Cocon L, Diaz O, Jacquemin C, et al. The current landscape of coronavirus-host protein–protein interactions. J Transl Med. 2020;18(1):1–15. 32811513 10.1186/s12967-020-02480-z PMC7432461

[83] Wang M, Li J, Cai J, et al. Overexpression of MicroRNA-16 alleviates atherosclerosis by inhibition of inflammatory pathways. Biomed Res Int. 2020;2020:8504238. 32775445 10.1155/2020/8504238 PMC7391121

[84] Yang Y, Yang F, Yu X, et al. miR-16 inhibits NLRP3 inflammasome activation by directly targeting TLR4 in acute lung injury. Biomed Pharmacother. 2019;112:108664. 30784935 10.1016/j.biopha.2019.108664

[85] Möhnle P, Hirschberger S, Hinske LC, et al. MicroRNAs 143 and 150 in whole blood enable detection of T-cell immunoparalysis in sepsis. Mol Med. 2018;24(1):54. 30332984 10.1186/s10020-018-0056-z PMC6191918

[86] Jaguszewski M, Osipova J, Ghadri J-R, et al. A signature of circulating microRNAs differentiates takotsubo cardiomyopathy from acute myocardial infarction. Eur Heart J. 2014;35(15):999–1006. 24046434 10.1093/eurheartj/eht392 PMC3985061

[87] Yang J, Yang X-S, Fan S-W, et al. Prognostic value of microRNAs in heart failure: a meta-analysis. Medicine (Baltimore). 2021;100(46):e27744. 34797300 10.1097/MD.0000000000027744 PMC8601330

[88] Telkoparan-Akillilar P, Cevik D. Identification of miR-17, miR-21, miR-27a, miR-106b and miR-222 as endoplasmic reticulum stress-related potential biomarkers in circulation of patients with atherosclerosis. Mol Biol Rep. 2021;48(4):3503–3513. 33860430 10.1007/s11033-021-06352-7

[89] Kim WR, Park EG, Kang K-W, et al. Expression analyses of MicroRNAs in hamster lung tissues infected by SARS-CoV-2. Mol Cells. 2020;43(11):953–963. 33199671 10.14348/molcells.2020.0177 PMC7700842

[90] Zheng C, Zheng Z, Sun J, et al. MiR-16-5p mediates a positive feedback loop in EV71-induced apoptosis and suppresses virus replication. Sci Rep. 2017. DOI:10.1038/s41598-017-16616-7. PMC5703983 29180670

[91] Farr RJ, Rootes CL, Rowntree LC, et al. Altered microRNA expression in COVID-19 patients enables identification of SARS-CoV-2 infection. PLoS Pathog. 2021;17(7):e1009759. 34320031 10.1371/journal.ppat.1009759 PMC8318295

[92] Chow JT-S, Prediction SL. Analysis of SARS-CoV-2-targeting MicroRNA in human lung epithelium. Genes (Basel). 2020;11. DOI:10.3390/genes11091002 PMC7565861 32858958

[93] Li C, Hu X, Li L, et al. Differential microRNA expression in the peripheral blood from human patients with COVID-19. J Clin Lab Anal. 2020;34(10):e23590. 32960473 10.1002/jcla.23590 PMC7536972

[94] Wang Z, Ruan Z, Mao Y, et al. miR-27a is up regulated and promotes inflammatory response in sepsis. Cell Immunol. 2014;290(2):190–195. 25043848 10.1016/j.cellimm.2014.06.006

[95] Chauhan N, Jaggi M, Chauhan SC, et al. COVID-19: fighting the invisible enemy with microRNAs. Expert Rev Anti Infect Ther. 2021;19(2):137–145. 32814446 10.1080/14787210.2020.1812385 PMC7870525

[96] Chen B, Han J, Chen S, et al. MicroLet-7b regulates neutrophil function and dampens neutrophilic inflammation by suppressing the canonical TLR4/NF-κB pathway. Front Immunol. 2021;12:653344. 33868293 10.3389/fimmu.2021.653344 PMC8044834

[97] Xie C, Chen Y, Luo D, et al. Therapeutic potential of C1632 by inhibition of SARS-CoV-2 replication and viral-induced inflammation through upregulating let-7. Signal Transduct Target Ther. 2021;6(1):84. 33619243 10.1038/s41392-021-00497-4 PMC7897876

[98] Katze MG, He Y, Jr GM. Viruses and interferon: a fight for supremacy. Nat Rev Immunol. 2002;2(9):675–687. 12209136 10.1038/nri888

[99] Mitash N, Donovan E, Swiatecka-Urban A J. The role of MicroRNA in the airway surface liquid homeostasis. Int J Mol Sci. 2020 21;21(11):3848. 10.3390/ijms21113848 PMC7312818 32481719

[100] Gracias DT, Stelekati E, Hope JL, et al. The microRNA miR-155 controls CD8(+) T cell responses by regulating interferon signaling. Nat Immunol. 2013;14(6):593–602. 23603793 10.1038/ni.2576 PMC3664306

[101] Garg A, Seeliger B, Derda AA, et al. Circulating cardiovascular microRNAs in critically ill COVID −19 patients. Eur J Heart Fail. 2021;23(3):468–475. 33421274 10.1002/ejhf.2096 PMC8014268

[102] Donyavi T, Bokharaei-Salim F, Baghi HB, et al. Acute and post-acute phase of COVID-19: analyzing expression patterns of miRNA-29a-3p, 146a-3p, 155-5p, and let-7b-3p in PBMC. Int Immunopharmacol. 2021;97:107641. 33895478 10.1016/j.intimp.2021.107641 PMC8023203

[103] Tacke F, Spehlmann ME, Vucur M, et al. miR-155 predicts long-term mortality in critically Ill patients younger than 65 years. Mediators Inflamm. 2019;2019:6714080. 30918471 10.1155/2019/6714080 PMC6409014

