# Supplementary figures and images for: Thrombosis-related circulating miR-16-5p is associated with disease severity in patients hospitalised for COVID-19
Source: RNA Biol. 2022 Aug 7;19(1):963–79. doi: 10.1080/15476286.2022.2100629 (PMC9361765; doi:10.1080/15476286.2022.2100629)

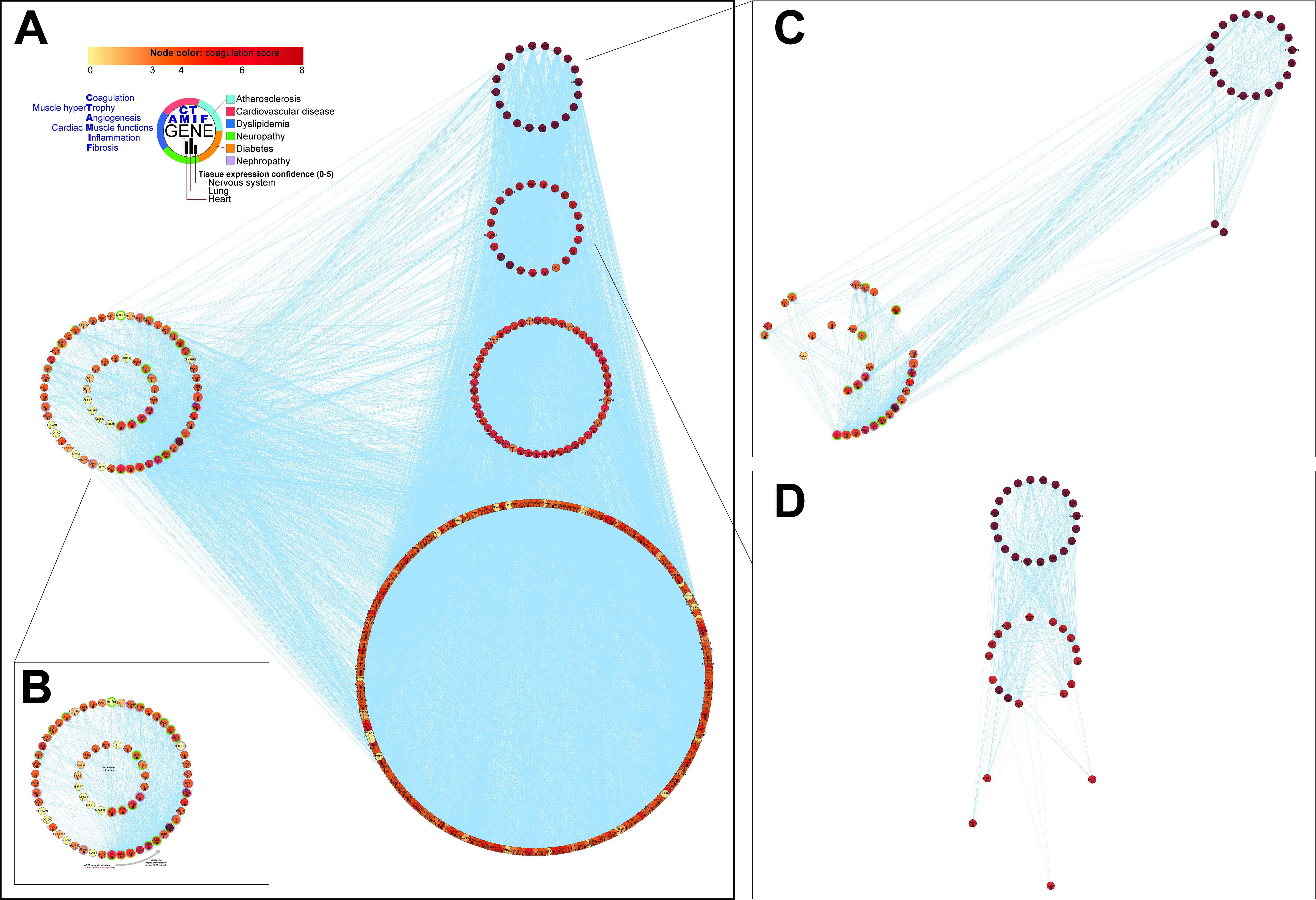

Supplement: Supplemental Material [file KRNB_A_2100629_SM0727.zip › Supp figure 1.tif]

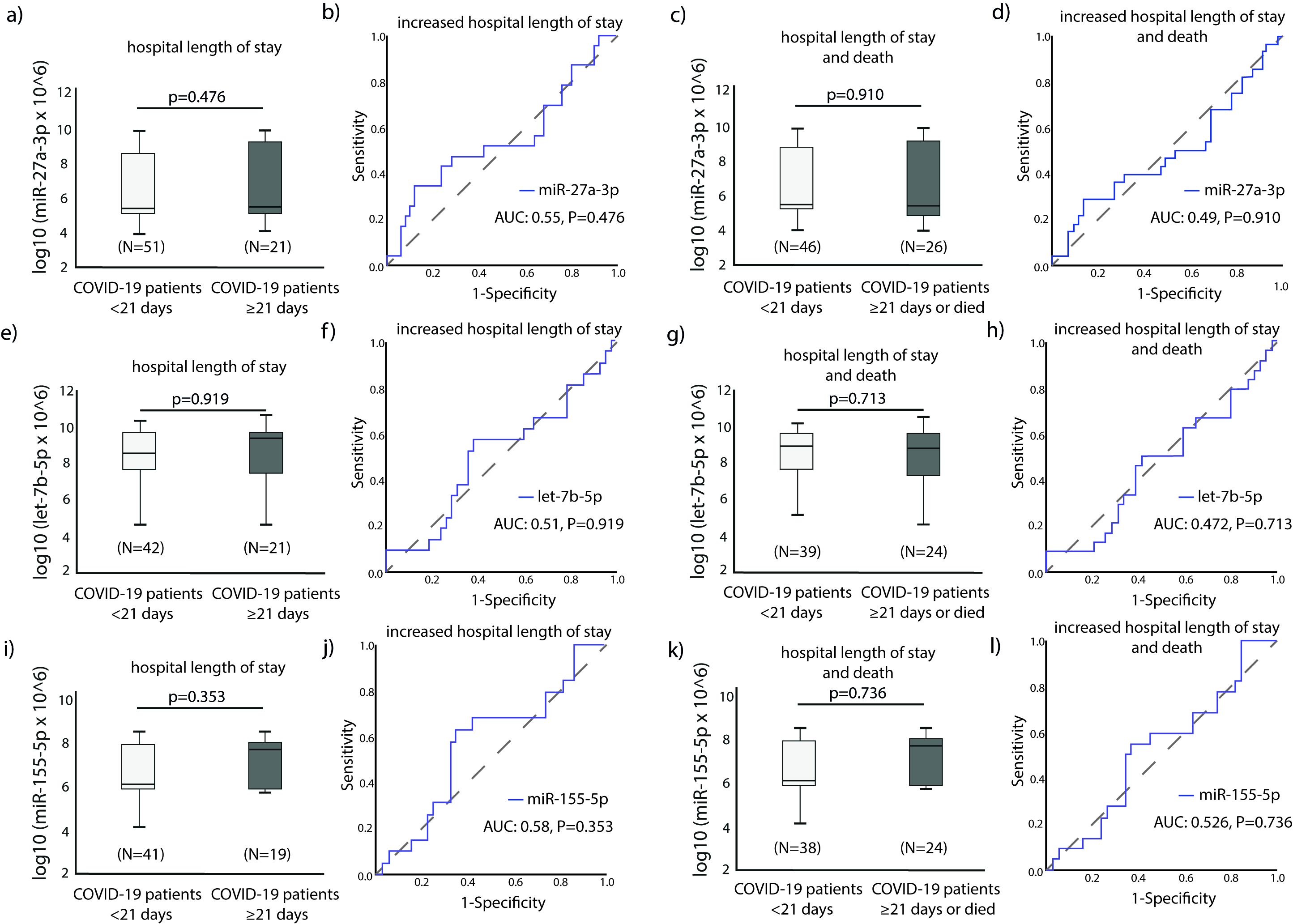

Supplement: Supplemental Material [file KRNB_A_2100629_SM0727.zip › supple figure 2 major revision.tif]
